# Supplementary material for: Inverse design of structural colours in polymeric films with crystallization-induced reversible thermochromism
Source: Nat Commun. 2025 Nov 18;16:10040. doi: 10.1038/s41467-025-66015-0 (PMC12627590; doi:10.1038/s41467-025-66015-0)
Supplement: Supplementary file 1 — Supplementary Information [file 41467_2025_66015_MOESM1_ESM.pdf]

Supplementary Information

Inverse design of structural colours in polymeric films with crystallization-induced reversible thermochromism

Dong Yang[1], Heyi Liang[2,], Chengjie Zhang[1], Peipei Shao[3], Qin Li[1], Yun Huang[1], Yi Dan[1], Cheng Zeng[4], Rui-Tao Wen[3], Long Jiang[1,*], Ming Xiao[1,*]*

[1]College of Polymer Science and Engineering, Polymer Research Institute, State Key Laboratory of Advanced Polymer Materials, Sichuan University, Chengdu, China.

[2]Pritzker School of Molecular Engineering, University of Chicago, Chicago, Illinois, United States.

[3]Department of Materials Science and Engineering, Southern University of Science and Technology, Shenzhen, China.

[4]Key Laboratory of Multifunctional Nanomaterials and Smart Systems, Suzhou Institute of Nano-Tech and Nano-Bionics, Chinese Academy of Sciences, Suzhou, China.

*Corresponding authors

E-mail: mingxiao@scu.edu.cn, jianglong@scu.edu.cn, heyi@uchicago.edu.cn

Table of Contents

Section 1. SS-SCF Model

Section 2. Multilayer Optical Model

Section 3. Synthesis Methods

Section 4. Characterization Methods

Section 5. Supplementary Tables and Figures

Section 1. SS-SCF Model

To predict domain spacing of self-assembled bottlebrush block copolymers (BBCPs) from their molecular structures, we extend the self-consistent field theory under strong segregation limit (SS-SCF) theoretical framework established by Zhulina *et al.* to BBCPs with distinct backbone and side chain chemistries.[1] This extension incorporates the influence of disparities between backbone and side chain on the conformation of bottlebrush polymers, as described by Liang *et al.*[2]

Section 1.1. Topological Ratio

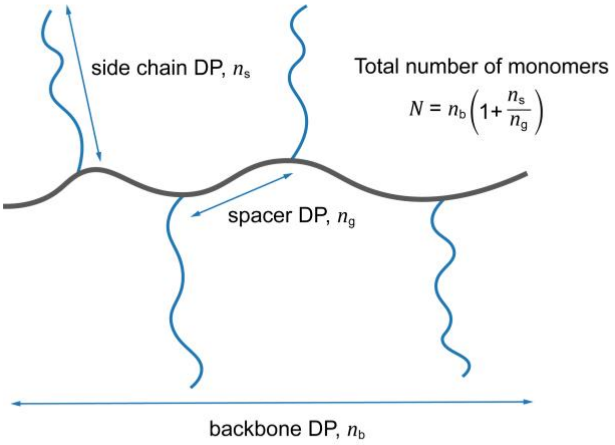

Supplementary Fig. 1. Scheme of a bottlebrush chain.

Consider a bottlebrush chain (Supplementary Fig. 1) consisting of a backbone with degree of polymerization (DP) n_b, and side chains with (DP) n_s evenly grafted on the backbone, and the DP of spacer between two adjacent grafting points is n_g. The total number of monomers in a bottlebrush chain, including the backbone and side chain monomers, is $N = n_b(1 + n_s/n_g)$. The monomer contour length (l_b and l_s), Kuhn length (b_b and b_s), and monomer volume (v_b and v_s) of the backbone and side chains are summarized in Table 1.

The free energy penalty of stretching the bottlebrush chain is:

$$\frac{F_{elastic}}{k_B T} \approx \frac{H^2}{R_0^2} = \frac{H^2\eta^2}{N b_b l_b} = \frac{H^2}{n_b b_K l_b}, \quad (1)$$

where R_0 and H are the end-to-end distance of the backbone before and after stretching, b_K is the effective Kuhn length of the bottlebrush.[1] The topological ratio η describes the effect of the chain architecture on the stretching free energy penalty and is defined as:

$$\eta^2 \equiv \frac{b_b}{b_K}\frac{N}{n_b} = \frac{b_b}{b_K}\left(1 + \frac{n_s}{n_g}\right). \tag{2}$$

The effective Kuhn length of bottlebrushes can be written as:

$$b_K = \frac{\Phi}{\Phi^*} b_b, \tag{3}$$

where Φ is the crowding parameter characterizing the mutual penetration between bottlebrushes, and Φ^* is the critical crowding parameter, which depends on the chemical structure of the bottlebrush and is on the order of unity.[2] For all bottlebrushes in this study, $n_s > b_b^2/b_s l_s$. Under such a condition, the crowding parameter is computed by:

$$\Phi = \frac{v_b}{(l_s b_s)^{1/2} l_b b_b} \frac{1}{n_s^{1/2}}\left(1 + \frac{v_s n_s}{v_b n_g}\right). \tag{4}$$

Eqs. (2-4) can be further simplified when $v_s n_s \gg v_b n_g$ and $n_s \gg n_g$:

$$\Phi \cong \frac{v_s}{(l_s b_s)^{1/2} l_b b_b} \frac{n_s^{1/2}}{n_g}, \tag{5}$$

$$b_K = \frac{\Phi}{\Phi^*} b_b \cong \frac{1}{\Phi^*} \frac{v_s}{(l_s b_s)^{1/2} l_b} \frac{n_s^{1/2}}{n_g}, \tag{6}$$

$$\eta^2 = \frac{b_b}{b_K}\left(1 + \frac{n_s}{n_g}\right) \cong \frac{\Phi^*}{\Phi}\frac{n_s}{n_g} = \Phi^* \frac{(l_s b_s)^{1/2} l_b b_b}{v_s} n_s^{1/2}. \tag{7}$$

For a bottlebrush block copolymer with A and B blocks, the ratio of the topological ratios between two blocks is:

$$\frac{\eta_B}{\eta_A} = \left(\frac{\Phi_\mathrm{B}^*}{\Phi_\mathrm{A}^*}\right)^{1/2} \left(\frac{n_{s,B} l_{s,B} b_{s,B}}{n_{s,A} l_{s,A} b_{s,A}}\right)^{1/4} \left(\frac{v_{s,B}}{v_{s,A}}\right)^{-1/2} \left(\frac{l_{b,B} b_{b,B}}{l_{b,A} b_{b,A}}\right)^{1/2} \tag{8}$$

Section 1.2. Free Energy of a Bottlebrush Block Copolymer Chain

Inspired by the framework of Zhulina *et al*,[1] we account for the differences between the backbone and side chains. Consider different superstructures indicated by the index i, where blocks A and B form lamella ($i = 1$), or the block A forms cylindrical ($i = 2$) or spherical ($i = 3$) domains embedded in the matrix of the block B. The domain spacing is d_i, the A domain size is R_i (i.e., the radius of a spherical or cylindrical domain, or the half-thickness of a lamella), the B domain size is $D_i = d_i/2 - R_i$. The elastic energy of block A is:

$$\frac{F_A^{(i)}}{k_B T} = \frac{R_i^2}{N_A l_{b,A} b_{b,A}} \eta_A^2 b_i \,, \tag{9}$$

with

$$b_i = \begin{cases} \pi^2/8, & i = 1 \\ \pi^2/16, & i = 2 \\ 3\pi^2/80, & i = 3 \end{cases} . \tag{10}$$

The elastic energy of block B is:

$$\frac{F_B^{(i)}}{k_B T} = \frac{R_i^2}{N_B l_{b,B} b_{b,B}} \eta_B^2 x g_i(x) = \frac{R_i^2}{N_A l_{b,A} b_{b,A}} \eta_B^2 g_i(x) \beta, \tag{11}$$

with

$$g_i(x) = \frac{\pi^2}{8} \begin{cases} x, & i = 1 \\ 6x^{-2}\left[(1+x)^{1/2} - 1\right]^4 \left[\frac{1}{4} + \frac{1}{3}\left[(1+x)^{1/2} - 1\right]^{-1}\right], & i = 2 \\ 9x^{-2}\left[(1+x)^{1/3} - 1\right]^5 \left[\frac{1}{5} + \frac{1}{2}\left[(1+x)^{1/3} - 1\right]^{-1} + \frac{1}{3}\left[(1+x)^{1/3} - 1\right]^{-2}\right], & i = 3 \end{cases} \tag{12}$$

where the ratio x and β are defined as:

$$x \equiv \frac{V_B}{V_A} \,, \tag{13}$$

$$\beta \equiv \frac{N_A l_{b,A} b_{b,A}}{N_B l_{b,B} b_{b,B}} x \,, \tag{14}$$

where V_α is the molecular volume of block A or B, with $\alpha = A$ or B:

$$V_\alpha = n_{b,\alpha} v_{b,\alpha} + n_{b,\alpha} \frac{n_{s,\alpha}}{n_{g,\alpha}} v_{s,\alpha} = n_{b,\alpha} v_{b,\alpha} \left(1 + \frac{v_{s,\alpha}}{v_{b,\alpha}} \frac{n_{s,\alpha}}{n_{g,\alpha}}\right). \tag{15}$$

For all bottlebrushes in this study, $v_s n_s \gg v_b n_g$ and $n_s \gg n_g$, so the molecular volume can be simplified as:

$$V_\alpha \cong v_{s,\alpha} n_{b,\alpha} \frac{n_{s,\alpha}}{n_{g,\alpha}} \cong v_{s,\alpha} N_\alpha. \tag{16}$$

Eqs. (13-14) can be simplified as:

$$x = \frac{V_B}{V_A} \cong \frac{N_B v_{s,B}}{N_A v_{s,A}} \,, \tag{17}$$

$$\beta = \frac{N_A l_{b,A} b_{b,A}}{N_B l_{b,B} b_{b,B}} x \cong \frac{v_{s,B} l_{b,A} b_{b,A}}{v_{s,A} l_{b,B} b_{b,B}} \,, \tag{18}$$

Therefore, the topological parameter defined by Eq. (31) in the literature[1] becomes:

$$\beta^{1/2}\frac{\eta_B}{\eta_A}=\left(\frac{v_{s,B}l_{b,A}b_{b,A}}{v_{s,A}l_{b,B}b_{b,B}}\right)^{1/2}\left(\frac{\Phi_{\mathrm{B}}^*}{\Phi_{\mathrm{A}}^*}\right)^{1/2}\left(\frac{n_{s,B}l_{s,B}b_{s,B}}{n_{s,A}l_{s,A}b_{s,A}}\right)^{1/4}\left(\frac{v_{s,B}}{v_{s,A}}\right)^{-1/2}\left(\frac{l_{b,B}b_{b,B}}{l_{b,A}b_{b,A}}\right)^{1/2}$$

$$=\left(\frac{\Phi_{\mathrm{B}}^*}{\Phi_{\mathrm{A}}^*}\right)^{1/2}\left(\frac{n_{s,B}l_{s,B}b_{s,B}}{n_{s,A}l_{s,A}b_{s,A}}\right)^{1/4}, \qquad (19)$$

The interfacial area per chain is:

$$s=\frac{iV_A}{R_i}=v_{s,A}n_{b,A}\frac{n_{s,A}}{n_{g,A}}=\frac{iv_{s,A}N_A}{R_i}\ . \qquad (20)$$

So, the surface free energy per chain is:

$$\frac{F_s^{(i)}}{k_BT}=\frac{iv_{s,A}N_A\gamma}{l_{b,A}^2R_i}\ , \qquad (21)$$

where $\gamma=\gamma_{A/B}l_{b,A}^2/k_BT$ is the dimensionless surface tension, i.e., surface free energy per area $l_{b,A}^2$ in unit of the thermal energy k_BT, and $\gamma_{A/B}$ is the interfacial tension between phases A and B.

The total free energy per chain is:

$$\frac{F^{(i)}}{k_BT}=\frac{F_A^{(i)}+F_B^{(i)}+F_s^{(i)}}{k_BT}=\frac{R_i^2}{N_Al_{b,A}b_{b,A}}\eta_A^2b_i+\frac{R_i^2}{N_Al_{b,A}b_{b,A}}\eta_B^2g_i(x)\beta+\frac{iv_{s,A}N_A\gamma}{l_{b,A}^2R_i}$$

$$=\frac{R_i^2}{N_Al_{b,A}b_{b,A}}\Psi_i(x)+\frac{iv_{s,A}N_A\gamma}{l_{b,A}^2R_i}\ . \qquad (22)$$

where the topology-dependent function is introduced:

$$\Psi_i(x)=\eta_A^2b_i+\eta_B^2g_i(x)\beta=\eta_A^2\left(b_i+g_i(x)\beta\frac{\eta_B^2}{\eta_A^2}\right)\ . \qquad (23)$$

Section 1.3. Domain Spacing

Minimize $F^{(i)}$ with respect to R_i, we get:

$$R_i=\left(\frac{i\gamma N_A^2v_{s,A}b_{b,A}}{2\Psi_{\mathrm{i}}(x)l_{b,A}}\right)^{\frac{1}{3}}\ . \qquad (24)$$

Due to the packing condition of the superstructure, the B domain size is:

$$D_i=R_i\left((1+x)^{\frac{1}{i}}-1\right)\ . \qquad (25)$$

Combining Eqs. (7) and (23-25), we can get the domain spacing:

$$d_i = 2(D_i + R_i) = 2R_i(1+x)^{1/i} = \left(4\frac{i\gamma N_A^2 v_{s,A} b_{b,A}}{\Psi_\mathrm{i}(x) l_{b,A}}\right)^{1/3} (1+x)^{1/i}$$

$$= \left(\frac{4\gamma v_{s,A} b_{b,A}}{\eta_A^2 l_{b,A}}\right)^{1/3} N_A^{2/3} i^{1/3} \left(b_i + g_i(x)\beta\frac{\eta_B^2}{\eta_A^2}\right)^{-1/3} (1+x)^{1/i}$$

$$= \left(\frac{4v_{s,A}^2}{\Phi_\mathrm{A}^*\left(n_{s,A} l_{s,A} b_{s,A}\right)^{1/2}} \frac{\gamma_{A/B}}{k_B T}\right)^{1/3} N_A^{2/3} i^{1/3} \left(b_i + g_i(x)\beta\frac{\eta_B^2}{\eta_A^2}\right)^{-1/3} (1+x)^{1/i}$$

$$= \tilde{L}_A N_A^{2/3} i^{1/3} \left(b_i + g_i(x)\beta\frac{\eta_B^2}{\eta_A^2}\right)^{-1/3} (1+x)^{1/i}, \qquad (26)$$

where we introduce a characteristic length scale of block A, $\tilde{L}_A$, defined as:

$$\tilde{L}_A \equiv \left(\frac{4}{\Phi_\mathrm{A}^*} \frac{v_{s,A}^2}{\left(n_{s,A} l_{s,A} b_{s,A}\right)^{1/2}} \frac{\gamma_{A/B}}{k_B T}\right)^{1/3} \qquad (27)$$

$\tilde{L}_A$ depends on the interfacial tension between A and B phases as well as the chemical structure and degree of polymerization of side chains on block A. Since the interfacial tension and the critical crowding parameter are unknown, the parameter $\tilde{L}_A$ is adjusted to align the theoretical prediction of domain spacing with experimental measurements when the crystallizable side chains (e.g., PEO or PCL) are in the melt state. Specifically, we compute $m_\mathrm{thy} = N_A^{2/3} i^{1/3} \left(b_i + g_i(x)\beta\frac{\eta_B^2}{\eta_A^2}\right)^{-1/3} (1+x)^{1/i}$ with the molecular parameters listed in Table 1 and plot them against four measured domain spacing measurements (d_expt) that are obtained from the reflection spectra. The characteristic length scale $\tilde{L}_A$ is then extracted from the slope of the linear fit. This procedure ensures that $\tilde{L}_A$ is determined consistently from multiple experimental data points, accounting for both measured spacings and known polymer parameters.

With measured experimental domain spacings from reflectance spectra (Supplementary Fig. 11 for PDMS-*b*-PEO and Supplementary Fig. 19a for PDMS-*b*-PCL), we can determine that $\tilde{L}_A = 0.44$ nm for PDMS-*b*-PEO (A: PDMS, B: PEO; Supplementary Fig. 12a), and $\tilde{L}_A = 0.34$ nm for PDMS-*b*-PCL (A: PDMS, B: PCL; Supplementary Fig. 19b). These values are consistent with direct evaluations based on Eq. (27), which yields $\tilde{L}_A = 0.44$ nm at room temperature, given $n_{s,A} = 68$ and molecular parameters for PDMS in Table 1, and setting the critical crowding parameter to a typical value $\Phi_\mathrm{A}^* = 0.4$ (as used for PNB-*g*-PLA in the literature[3]), and the interfacial tension $\gamma_{A/B} = 10.6\ mN \cdot m^{-1}$(PDMS/PEO interface from the literature[4]).

It is challenging to predict the precise effect of crystallization on the change in domain spacing due to the difficulty of determining the exact chain conformation of crystallized side chains. For simplicity, we assume that the effect of crystallization can be captured through a combination of increased domain density and the stiffening of the side chains. Under these assumptions, the domain density can be calculated given the crystallinity and densities of melt and crystalline states, and the only undetermined parameter required to obtain the domain spacing in the crystalline state is the stiffening of side chains, defined as the ratio of the Kuhn lengths of the side chains in the crystalline and melt states, $\alpha_s \equiv b_{s,c}/b_{s,m}$. According to Eq. (26), the lamellar spacing d is a function of $\tilde{L}_{\mathrm{A}}$, η_{A} and η_{B} (with A = PDMS and B = PEO or PCL). Considering side chain A does not crystallize and side chain B is crystallizable, we can assume that $\tilde{L}_{\mathrm{A}}$ remains constant and the topological parameter ($\beta \frac{\eta_{\mathrm{B}}^2}{\eta_{\mathrm{A}}^2}$) will change with α_{s} based on Eq. (19). Therefore, variations in α_{s} lead to corresponding changes in domain spacing d.

To obtain the value of α_s, we use the value of $\tilde{L}_A$ obtained from the melt state into Eq. (26), i.e. $\tilde{L}_A = 0.44$ nm for PDMS-*b*-PEO and $\tilde{L}_A = 0.35$ nm for PDMS-*b*-PCL, and then minimize the mean squared error between theoretical prediction and experimental measurements (Supplementary Fig. 12b for PDMS-*b*-PEO and Supplementary Fig. 19d for PDMS-*b*-PCL). It is determined that $\alpha_s = 1.96$ for PDMS-*b*-PEO and $\alpha_s = 1.62$ for PDMS-*b*-PCL.

We can use a generalized formulation with ($\alpha_{s,A}$, $\alpha_{s,B}$) for copolymers where both blocks undergo crystallization or experience different degrees of stiffening, but in our specific case the single-parameter description is sufficient. Note that a single value α_s is introduced to phenomenologically describe the effect of side chain crystallization, with several assumptions: (1) the crystallinity is almost the same for all architectures; (2) the interfacial tension between two blocks is not affected by the crystallization; (3) the crystallization only involves the local reorganization of side chains and the formation of spherulites[5] is suppressed due to the strong confinement of the lamellar structure.

Section 1.4. Phase Diagram

Following the framework of Zhulina *et al*,[1] the transition between different superstructure is determined by comparing the free energy per chain $F^{(i)}$ in different morphologies (i = 1, 2, 3), as shown in Eq. (22). The boundary lines that separate the lamellar (L), cylindrical (C), and spherical (S) regimes are determined by $F^{(i)}(x) = F^{(i+1)}(x)$ (i = 1, 2). Combining Eqs. (22) and (24), this condition reduces to:

$$i^2\Psi_i(x) = (i+1)^2\Psi_{i+1}(x), \quad i = 1, 2\,. \tag{28}$$

Substituting $\Psi_i(x)$ with Eq. (23), we obtain the boundary lines in the phase diagram:

$$\beta^{1/2}\frac{\eta_B}{\eta_A}=\begin{cases}\sqrt{(4b_2-b_1)/[g_1(x)-4g_2(x)]} & \mathrm{L-C}\\ \sqrt{(9b_3-4b_2)/[4g_2(x)-9g_3(x)]} & \mathrm{C-S}\\ \sqrt{[g_1(1/x)-4g_2(1/x)]/(4b_2-b_1)} & \mathrm{L-}C'\\ \sqrt{[4g_2(1/x)-9g_3(1/x)]/(9b_3-4b_2)} & \mathrm{C'-S'}\end{cases},\quad (29)$$

where C' and S' are inverted cylindrical and spherical regimes with the block A forming the matrix. Therefore, the phase diagram for bottlebrush diblock copolymers can be constructed in $\beta^{1/2}\frac{\eta_B}{\eta_A}$ *vs.* f_B, where the volume fraction of the B block is $f_B = V_B/(V_A+V_B) = x/(1+x)$.

Section 2. Multilayer Optical Model

To find the thickness of each layer in BBCP photonic films from the reflectance spectrum, we use a multilayer optical model. Using a transfer matrix method, we present each layer in a multilayer structure as a matrix that describes the relationship between the electric fields of incident, reflected, and transmitted light at the layer boundaries. By calculating the transfer matrices for each layer, and multiplying them together, we can determine the overall reflectance and transmittance of the multilayer structure. This method accounts for interference and reflection at each interface, allowing for accurate prediction of the optical response across various wavelengths and angles.

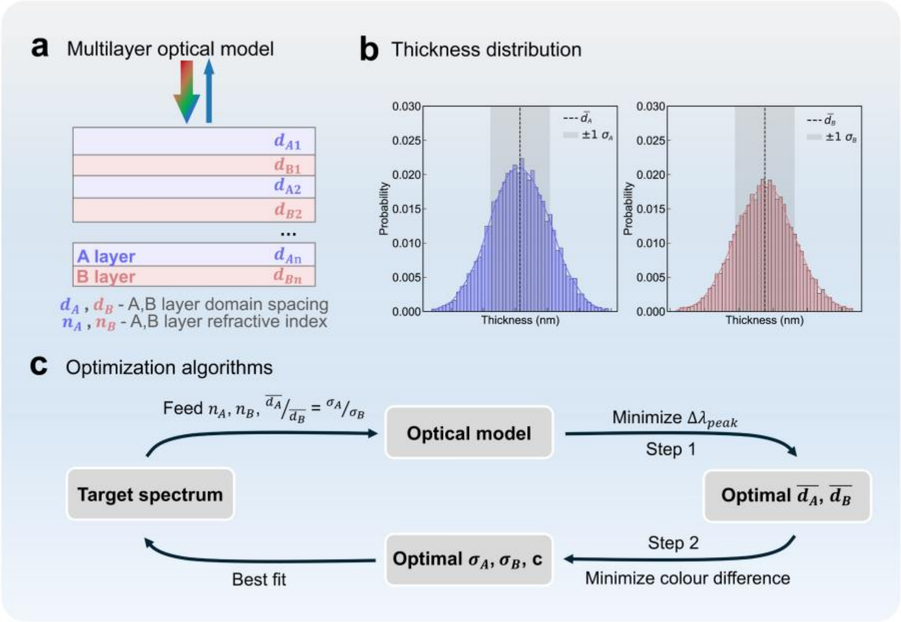

Supplementary Fig. 2. The modified multilayer optical model that integrates Bayesian optimization. **a,** Diagram to show alternating A/B layer structure in the optical model. **b,** Normal distribution of A and B

layer thicknesses **c,** Flowchart illustrating the parameter optimization process of the optical model using Bayesian optimization.

In the BBCP photonic films, we alternate block A and B layers to form a multilayer structure. The thickness ratio of each A and B layer is equal to the volume ratio between two blocks. We incorporate angular variation scans from 0° to 24° with 8° step intervals, which is consistent with the measurement angle range that is defined by the objective (NA = 0.21) in the customized microspectrometer. The lamellar spacing is calculated from the reflectance spectra obtained with this measurement method. The refractive index of each layer can obtained either based on ellipsometer data and Bruggeman approximation or from literature.[6]

The layers in BBCP films have noticeable variations in thickness. To model the thickness inhomogeneity, we assume the thickness of each layer (d) to follow a truncated normal distribution,

$$p(d) = \frac{\frac{1}{\sqrt{2\pi\sigma^2}} e^{-\frac{(d-\bar{d})^2}{2\sigma^2}}}{\Phi(-3) - \Phi(3)}, \tag{30}$$

where the mean values, $\overline{d_A}$ and $\overline{d_B}$, are set to predefined values, with the standard deviation σ_A and σ_B representing the degree of experimental variations in thickness. The truncation points are set to the mean values $\pm$ three standard deviations to prevent the thickness from becoming unphysically large or too small, in accordance with the "3-sigma rule" of normal distribution. Φ is the cumulative distribution function (CDF) of the standard normal distribution, representing the probability that a standard normal random variable takes a value no greater than x:

$$\Phi(x) = \int_{-\infty}^{x} \frac{1}{\sqrt{2\pi}} e^{-t^2/2} \, dt. \tag{31}$$

We generate two sets of random numbers $[d_{A1}, d_{A2}, d_{A3}, \ldots, d_{An}]$, $[d_{B1}, d_{B2}, d_{B3}, \ldots, d_{Bn}]$ based on the distribution functions to simulate the block thicknesses (Supplementary Fig. 2b), and then arrange them sequentially to form a multilayer structure. This random approach can lead to inconsistent internal thickness distributions, resulting in different spectra from the optical model. To address this issue, we generate multiple thickness distributions $[d_{A1}, d_{A2}, d_{A3}, \ldots, d_{An}]_1$, $[d_{A1}, d_{A2}, d_{A3}, \ldots, d_{An}]_2, \ldots$, $[d_{A1}, d_{A2}, d_{A3}, \ldots, d_{An}]_i$ $(i = 200$ in our case) in a single simulation and then average the reflectance (R) over these distributions, $R = \frac{1}{i}\sum_{k=1}^{i} R_k$.

To consider the background effect in the reflectance spectrum, we add a parameter c to the calculated intensity. This establishes a complete process for calculating spectra

from experimental parameters $[\overline{d_A}, \overline{d_B}, \sigma_A, \sigma_B, c]$, with randomness controlled within an acceptable range.

As shown in Supplementary Fig. 2c, we start with a target spectrum and use two steps of Bayesian optimization to find the optimal experimental parameters $[\overline{d_A}, \overline{d_B}, \sigma_A, \sigma_B, c]_{optimized}$. In the first step, we define the objective function as the difference between the peak wavelength of the calculated and target spectra. We use Bragg's law to estimate the thicknesses of two blocks, $\overline{d_A}$ and $\overline{d_B}$, which are then used as initial values for optimization. The ratio $\overline{d_A}/\overline{d_B}$ can be calculated from the volume ratios between two blocks in BBCP samples. We set σ_A and c to appropriate values (their values do not affect the position of the peak) to obtain the optimal $\overline{d_A}$. In the second step, we set the objective function as the colour difference between the calculated and target spectra to find optimal σ_A and c with fixing $\overline{d_A}$ obtained from the first step. Since the ratios of σ_A to σ_B and $\overline{d_A}$ to $\overline{d_B}$ are known, we only need to obtain the parameters for A to calculate those for B. We then obtain the optimal experimental parameters that minimize the colour difference. However, some discrepancy remains between the calculated and measured spectra. We attribute this difference to the fact that the optical model assumes flat, smooth, and uniform defect-free layers, whereas real samples contain curved layers with interfacial roughness likely caused by polymer dispersity and self-assembly defects.

For PDMS-*b*-PEO films, we can input the refractive index and the ratios of d_{PDMS} to d_{PEO} as inputs, and then determine the optimal values of $[\overline{d_{\mathrm{PDMS}}}, \overline{d_{\mathrm{PEO}}}, \sigma_{\mathrm{PDMS}}, \sigma_{\mathrm{PEO}}]$, via the aforementioned optimization method. These values are summarized in Supplementary Table 3.

Section 3. Synthesis Methods

Synthesis of *N*-(carboxyhexyl)-*cis*-5-norbornene-*exo*-2,3-dicarboxyimide (NBI-COOH)

We mixed *cis*-5-norbornene-*exo*-2,3-dicarboxylic anhydride (6.0 g, 36.6 mmol), 6-aminohexanoic acid (4.8 g, 36.6 mmol), triethylamine (514.3 mg, 3.7 mmol), and toluene (150 mL) in a 500 mL one-neck round-bottom flask equipped with a magnetic stirring bar, a Dean-Stark apparatus, and a reflux condenser. The mixture was stirred at 130°C for 16 hours. The solution was then cooled to room temperature, and the crude product was repeatedly washed with 1M HCl (aq.), dried over Na_2SO_4 (s.), and concentrated in vacuo to yield a brown solid. This solid was recrystallized with ethyl acetate to obtain NBI-COOH as white powders.[7]

Synthesis of PEO macromonomers (NBI-PEO)

We sequentially added NBI-COOH (0.7 g, 2.5 mmol), DMAP (61.1 mg, 0.5 mmol), and anhydrous dichloromethane (25 mL) into a 250 mL reaction flask. EDC·HCl (668.3 mg, 3.5 mmol) was slowly added at 0°C. After stirring for 15 minutes, monohydroxy-terminated PEO (4.67 g, 1.0 mmol) was added, and the mixture was stirred for 48 hours at room temperature. The reaction mixture was diluted with dichloromethane, washed with 1M HCl (aq.), saturated $NaHCO_3$ solution, water, and dried over Na_2SO_4 (s.).The product was concentrated under reduced pressure, then precipitated into diethyl ether to obtain NBI-PEO, followed by vacuum drying at 45°C for 24 hours.[8]

Synthesis of PDMS macromonomers (NBI-PDMS)

We sequentially added NBI-COOH (0.7 g, 2.5 mmol), DMAP (61.1 mg, 0.5 mmol), and anhydrous dichloromethane (25 mL) into a 250 mL reaction flask. EDC·HCl (668.3 mg, 3.5 mmol) was slowly added at 0°C. After stirring for 15 minutes, monohydroxy-terminated PDMS (4.67 g, 1.0 mmol) was added, and the mixture was stirred for 48 hours at room temperature. The reaction mixture was diluted with dichloromethane, washed with 1M HCl (aq.) and water, and dried over Na_2SO_4 (s.). The solution was passed through activated basic alumina. After solvent evaporation, the colourless liquid was dried under vacuum at 45°C for 24 hours to obtain NBI-PDMS.[9]

Synthesis of PCL macromonomers (NB-PCL)

Typically, 5-norbornene-2-methanol (0.415 g, 3.25 mmol), ε-caprolactone (10 g, 87.7 mmol), and $Sn(Oct)_2$ (0.05 g) in a 100 mL Schlenk flask was subjected to three freeze-pump-thaw cycles. The reaction mixture was magnetically stirred at 100°C for 8 hours under N_2 atmosphere. The reaction product was dissolved in THF (25 mL), then precipitated into methanol to obtain NB-PCL, followed by vacuum drying at 45°C for 24 hours.[8]

Synthesis of PS macromonomers (NBI-PS)

We synthesized NBI-PS using the Atom Transfer Radical Polymerization (ATRP) method.[10] First, We sequentially added *N*-(hydroxyethyl)-*cis*-5-norbornene-*exo*-2,3-dicarboximide (5.02 g, 24.2 mmol), triethylamine (4.6 mL, 32 mmol) and anhydrous dichloromethane (160 mL) into in a 500 mL reaction flask. 2-Bromoisobutyrylbromide (4.4 mL, 36 mmol) was then slowly added at 0°C. After stirring for 20 minutes, the mixture was stirred for 20 hours at room temperature. The reaction mixture was washed with 1M HCl (aq.), saturated $NaHCO_3$ (aq.), brine and then dried over Na_2SO_4 (s.) and concentrated in vacuo. The crude product was purified by silica gel chromatography to give the final product (initiator NBI-Br).

Styrene (12 mL, 0.105 mol) was passed through basic aluminum oxide to remove inhibitors and then added to a dried 100 mL Schlenk flask. CuBr (35 mg, 0.27 mmol) and initiator NBI-Br (335.2 mg, 0.94 mmol) were subjected to three freeze-pump-thaw cycles. PMDETA (54 μL, 0.26 mmol) was then added to the mixture followed by additional consecutive freeze-pump-thaw cycles and the solution stirred for 5 minutes. The reaction mixture was stirred at 100°C for 3 hours under N_2 atmosphere. Subsequently, it was rapidly cooled with liquid nitrogen and dissolved in THF (25 mL). The resulting solution was then passed through basic alumina to remove the copper catalyst and precipitated in methanol, dried for 24 hours at 45°C.

Block bottlebrush copolymers (BBCPs) including PDMS-*b*-PEO, PDMS-*b*-PCL, and PS-*b*-PCL were synthesized by sequential ring-opening metathesis polymerization (ROMP) in a glovebox under anhydrous conditions. In a typical procedure, the first monomer (NBI-functionalized macromonomer, 100 mg) was dissolved in anhydrous THF (2 mL) in a 10 mL vial with stirring. The third-generation Grubbs catalyst (G3) was prepared in THF at a concentration of 2.0 mg mL^{-1} and added quickly with rapid stirring, using a calculated volume based on the desired degree of polymerization. After stirring at room temperature (10-25 min depending on monomer system), the second monomer solution (NB-PEO_{105}, NB-PCL_{38}, or NB-PCL_{22}) was added and allowed to react for 4 hours. The reaction was quenched with ethyl vinyl ether. The resulting polymers were further purified by precipitation into methanol to obtain white solid products.

Section 4. Characterization Methods

Characterization of BBCPs' molecular weight. To determine the relative molecular weight of macromonomers and bottlebrush homopolymers, we used GPC (Waters 1515) containing three Styragel HR 300 × 7.8 mm^2 columns connected with a refractive index

(RI) detector using polystyrene standards (American Polymer Standards Corp.). THF was used as the eluent with a flow rate of 1.0 mL min^{-1} at 25°C.

Characterization of statical optical properties. We measured the crystallinity of BBCP films using an X-ray diffractometer (XRD) (Rigaku, Ultima IV, Japan, λ = 0.154 nm) and scans at 3° min^{-1} in the 2θ range from 5° to 80°.

Characterization of dynamic optical properties. We took dynamic spectra and microscopic images during heating-cooling cycles using an optical microscope coupled with a thermal stage (PE-35130, Suzhou Keyiqian Precision Equipment Co., Ltd.) and a fiber-optic spectrometer (Ideaoptics PG2000-PRO-EX). Considering there were temperature differences between the upper and lower surfaces, we attached a thermocouple to the sample surface and recorded it as *in-situ* temperature. Reflectance spectra were obtained using a PTFE diffuse reflector sheet (Thorlabs, PMR10P1) as a white reference. The reflectance spectra were smoothed using a rolling window method with a window size of 10. In addition, the melting and crystallization process of BBCPs was imaged using a polarizing microscope (Nikon LV100NPOL) coupled with a thermal stage (Linkam THMS600).

Refractive index measurement. To measure the refractive index of BBCPs, we used an ultraviolet-near-infrared (UV-NIR) ellipsometer (J.A.Woollam, M-2000DI) with a rotating compensator. BBCP films were prepared by spin coated on a silicon wafer. We collected the Psi (Ψ) and Delta (Δ) data versus wavelength (360-800 nm) at three incident angles, 65°, 70°, and 75° in reflection mode. All data were analyzed through the software J.A.Woollam CompleteEASE v6.0 to build models. Using a two-layer hybrid model of Cauchy and B-spline, we matched experimental data with calculation to determined corresponding optical constants and thickness. The quality of the fit was assessed through defined Mean Squared Error (MSE):

$$MSE_{NCS} = \sqrt{\frac{1}{3j-m}\sum_{i=1}^{j}\left[\left(\frac{N_{\mathrm{meas_i}} - N_{\mathrm{calc_i}}}{0.001}\right)^2 + \left(\frac{C_{\mathrm{meas_i}} - C_{\mathrm{calc_i}}}{0.001}\right)^2 + \left(\frac{S_{\mathrm{meas_i}} - S_{\mathrm{calc_i}}}{0.001}\right)^2\right]}, \quad (32)$$

where j was number of wavelengths, m was the number of fit parameters, $N = cos(2\Psi)$, $C = sin(2\Psi)cos(\Delta)$, $S = sin(2\Psi)sin(\Delta)$, and the superscripts "meas" and "calc" of the parameters represented measured and calculated ellipsometry results

We calculated the refractive index of PDMS block based on the Bruggeman effective medium approximation,[6]

$$\sum_i f_i\left(\frac{n_i^2 - n_{eff}^2}{n_i^2 + n_{eff}^2}\right) = 0\,, \quad (33)$$

where i represented the PDMS block or PCL block, f_i represented the volume fractions of i block, and n_i represented the refractive index of i block.

We calculated the volume fractions of PCL block (f_{PCL}) in synthesized BBCPs based on the density of each segment (0.97 g·cm^{-3} for PDMS, 1.25 g·cm^{-3} for PEO, 1.145 g·cm^{-3} for PCL and 1.05 g·cm^{-3} for PS).[11] Typically,

$$f_{\mathrm{PCL}} = \frac{V_{\mathrm{PCL}}}{V_{\mathrm{PCL}} + V_{\mathrm{PDMS}}} = \frac{m_{\mathrm{PCL}}/\rho_{\mathrm{PCL}}}{m_{\mathrm{PCL}}/\rho_{\mathrm{PCL}} + m_{\mathrm{PDMS}}/\rho_{\mathrm{PDMS}}}$$
$$= \frac{n_{b,\mathrm{PCL}} * M_{s,\mathrm{PCL}}/\rho_{\mathrm{PCL}}}{n_{b,\mathrm{PCL}} * M_{\mathrm{s,PCL}}/\rho_{\mathrm{PCL}} + n_{b,\mathrm{PDMS}} * M_{\mathrm{s,PDMS}}/\rho_{\mathrm{PDMS}}}, \qquad (34)$$

where V_{PCL}, V_{PDMS} represented the PCL and PDMS volumes, m_{PCL}, m_{PDMS} were experimental feeding mass, ρ_{PCL}, ρ_{PDMS} represented the density, $n_{b,\mathrm{PCL}}$, $n_{b,\mathrm{PDMS}}$ represented feeding moles, and $M_{\mathrm{s,PCL}}$, $M_{\mathrm{s,PDMS}}$ were the molecular weights of side chains of the PCL and PDMS blocks.

We estimated the peak shift during heating and cooling based on the Bragg's law,

$$\lambda_{\mathrm{heat}} = 2n_{\mathrm{heat}}d_{\mathrm{heat}} \sin\theta, \qquad (35)$$

$$\lambda_{\mathrm{cool}} = 2n_{\mathrm{cool}}d_{\mathrm{cool}} \sin\theta, \qquad (36)$$

$$\frac{\Delta\lambda}{\lambda_{\mathrm{cool}}} = \frac{\lambda_{\mathrm{heat}} - \lambda_{\mathrm{cool}}}{\lambda_{\mathrm{cool}}} = \frac{n_{\mathrm{heat}}d_{\mathrm{heat}}}{n_{\mathrm{cool}}d_{\mathrm{cool}}} - 1, \qquad (37)$$

where λ_{heat}, λ_{cool} represented the peak wavelengths, n_{heat}, n_{cool} represented the refractive index, and d_{heat}, d_{cool} represented the thickness of BBCP films after heating and cooling.

Section 5. Supplementary Tables and Figures

Table 1. Molecular parameters of backbones and side chains

	Polymer	Monomer contour length l [nm][a]	Kuhn length b [nm][b]	Monomer volume v [nm^3][c]	Density ρ [g/cm^3]
Back bone	PNB	0.61	1.18	0.153	1.35
	PNBI	0.61	1.18	0.341	1.35
Side chain	PDMS	0.29	1.22	0.127	0.97
	PEO	0.33	1.15	0.065	1.12
	PCL	0.86	0.78	0.189	1.15
	PCL[d]	0.15	0.64	0.027	1.15

[a] Monomer contour length is obtained by geometry optimization.

[b] Kuhn length is obtained for linear chain, calculated by $b = c_\infty n\langle l_{bond}^2\rangle/Nl = c_\infty n_{bond}\langle l_{bond}^2\rangle/l$, where n_{bond} is the number of bonds per monomer, N is the degree of polymerization (DP). Data for PNB is estimated based on literature.[12] PNBI and PNB are assumed to have the same Kuhn length. Kuhn lengths of PDMS and PEO are obtained from a textbook.[13] Kuhn length of PCL is obtained from literature.[14]

[c] Monomer volume is calculated by $v = M_0/(\rho\mathcal{N}_{Av})$, where M_0 is the molar mass of the monomer, ρ is the mass density, $\mathcal{N}_{Av}$ is the Avogadro's number.

[d] Regard one PCL monomer as seven equivalent small monomers.

Table 2. Molecular weight, dispersity, crystallinity, melting and crystallization temperatures of PDMS-*b*-PCL and PCL homo polymers.

Sample names	M_w (×10^6 g mol^{-1})	$Đ$	f_{PCL}(% v/v)	X_c(%)^a	T_m (°C)	T_c (°C)
P-PCL$_{22}$	0.468	1.18	100	45.54	43.91	8.090
P-PCL$_{29}$	0.839	1.17	100	49.07	48.16	14.06
P-PCL$_{38}$	1.22	1.20	100	44.93	50.66	16.88
PDMS$^{90}_{68}$-50%PCL$^{126}_{38}$	1.16	1.54	50	40.91	49.45	14.09
PDMS$^{110}_{68}$-50%PCL$^{155}_{38}$	1.86	1.25	50	40.81	49.75	15.40
PDMS$^{130}_{68}$-50%PCL$^{183}_{38}$	2.16	1.26	50	40.08	49.81	15.29
PDMS$^{150}_{68}$-50%PCL$^{210}_{38}$	2.36	1.24	50	40.57	49.57	15.55
PDMS$^{90}_{68}$-42%PCL$^{92}_{38}$	0.97	1.23	42	44.37	49.34	14.97
PDMS$^{110}_{68}$-42%PCL$^{113}_{38}$	1.40	1.26	42	45.11	49.33	15.39
PDMS$^{130}_{68}$-42%PCL$^{133}_{38}$	1.72	1.27	42	41.41	49.60	15.86
PDMS$^{150}_{68}$-42%PCL$^{154}_{38}$	1.79	1.24	42	44.04	49.54	15.83
PDMS$^{90}_{68}$-30%PCL$^{54}_{38}$	1.52	1.25	30	44.72	48.88	12.81
PDMS$^{110}_{68}$-30%PCL$^{66}_{38}$	2.12	1.26	30	43.52	49.57	17.14
PDMS$^{130}_{68}$-30%PCL$^{78}_{38}$	2.76	1.26	30	41.85	49.04	14.43
PDMS$^{150}_{68}$-30%PCL$^{90}_{38}$	3.28	1.26	30	40.58	49.28	15.49
PDMS$^{90}_{68}$-42%PCL$^{114}_{29}$	0.82	1.39	42	41.08	47.23	11.47
PDMS$^{110}_{68}$-42%PCL$^{139}_{29}$	0.92	1.34	42	44.73	48.01	13.89
PDMS$^{130}_{68}$-42%PCL$^{165}_{29}$	1.21	1.37	42	43.96	48.18	13.30
PDMS$^{150}_{68}$-42%PCL$^{190}_{29}$	1.39	1.42	42	36.77	47.73	11.28
PDMS$^{90}_{68}$-42%PCL$^{149}_{22}$	1.05	1.17	42	36.18	43.46	4.260
PDMS$^{110}_{68}$-42%PCL$^{182}_{22}$	1.49	1.21	42	35.20	43.32	4.930
PDMS$^{130}_{68}$-42%PCL$^{215}_{22}$	1.54	1.19	42	35.91	43.13	4.850
PDMS$^{150}_{68}$-42%PCL$^{248}_{22}$	2.12	1.22	42	35.72	43.78	5.760
PDMS$^{120}_{68}$-40%PCL$^{139}_{29}$	2.05	1.45	40	/	/	/
PDMS$^{120}_{68}$-50%PCL$^{208}_{29}$	2.36	1.39	50	/	/	/
PDMS$^{120}_{68}$-60%PCL$^{251}_{29}$	2.77	1.44	60	/	/	/
PDMS$^{135}_{68}$-38%PCL$^{190}_{22}$	1.99	1.38	38	/	/	/
PDMS$^{135}_{68}$-45%PCL$^{190}_{29}$	2.43	1.29	45	/	/	/
PDMS$^{135}_{68}$-50%PCL$^{190}_{38}$	2.52	1.23	50	/	/	/

The crystallinity (X_c) is calculated using the equation, $X_c = \left[\Delta H(T_m)/\left(\Delta H(T_m^0) * w(PCL)\right)\right] * 100\%$, where $\Delta H(T_m)$ is the enthalpy change measured at the melting point and $w(PCL)$ is the PCL mass fraction. $\Delta H(T_m^0)$ is the enthalpy change of a completely crystalline sample, which is $\Delta H(T_m^0) = 139.3$ J g^{-1} obtained from the literature.[15]

Table 3 The optimal values of $\left[\overline{d_{\mathrm{PDMS}}}, \overline{d_{\mathrm{PEO}}}, \sigma_{\mathrm{PDMS}}\right]$ in the multilayer film optical model and dispersity of PDMS-*b*-PEO samples.

Sample names	$Đ$	$\overline{d_{\mathrm{PDMS}}}$ (nm)	$\overline{d_{\mathrm{PEO}}}$ (nm)	ΔE^{a}	σ_{PEO}
PDMS$^{90}_{68}$-46%PEO$^{79}_{105}$	1.31	89.3	76.1	13.4	9.1
PDMS$^{100}_{68}$-46%PEO$^{88}_{105}$	1.31	96.2	82.0	9.4	9.5^{b}
PDMS$^{110}_{68}$-46%PEO$^{97}_{105}$	1.34	108.0	92.0	7.6	12.1
PDMS$^{120}_{68}$-46%PEO$^{106}_{105}$	1.39	118.0	100.5	2.8	16.3^{b}
PDMS$^{130}_{68}$-46%PEO$^{114}_{105}$	1.41	126.9	108.1	7.5	18.4
PDMS$^{150}_{68}$-46%PEO$^{132}_{105}$	1.44	144.9	123.4	7.7	23.9

^{a} The colour difference between the calculated and target spectra. ^{b} The standard deviation (σ) of the designed data thickness was obtained through a linear equation relating the polydispersity index (PDI) to the standard deviation of thickness distribution.

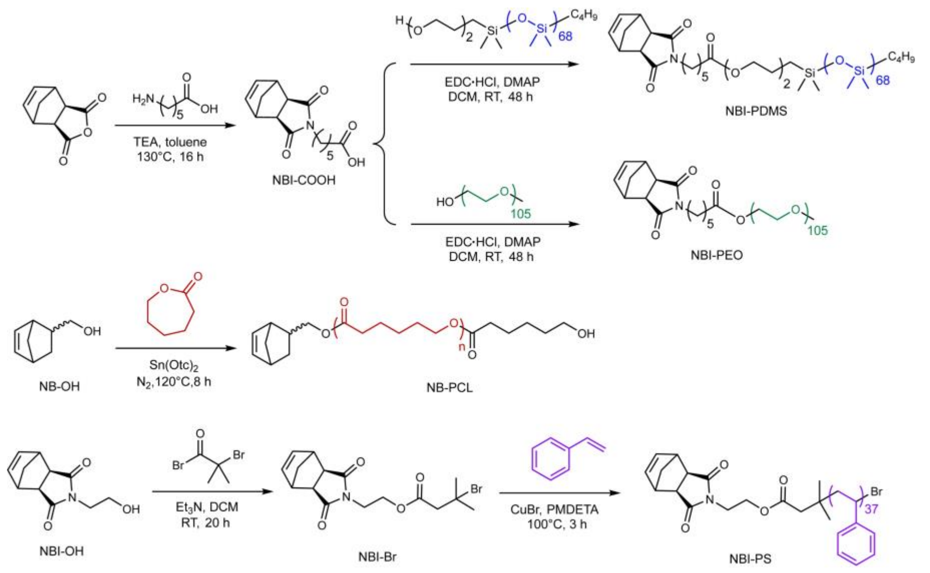

Supplementary Fig. 3. **Synthetic routes of macromonomers.** Schematic illustration showing the synthetic procedures for four norbornene-functionalized macromonomers. NBI-PDMS and NBI-PEO were obtained through an EDC/DMAP-catalyzed esterification between NBI-COOH and monohydroxy-terminated PDMS or monohydroxy-terminated PEO. NBI-PCL was prepared by $Sn(Oct)_2$-catalyzed Ring-Opening Polymerization (ROP) of ε-caprolactone initiated with 5-norbornene-2-methanol. NBI-PS was synthesized by Atom Transfer Radical Polymerization (ATRP) using a norbornene-based initiator (NBI-Br). These macromonomers carry reactive norbornene groups that enable their polymerization via ROMP to form bottlebrush block copolymers.

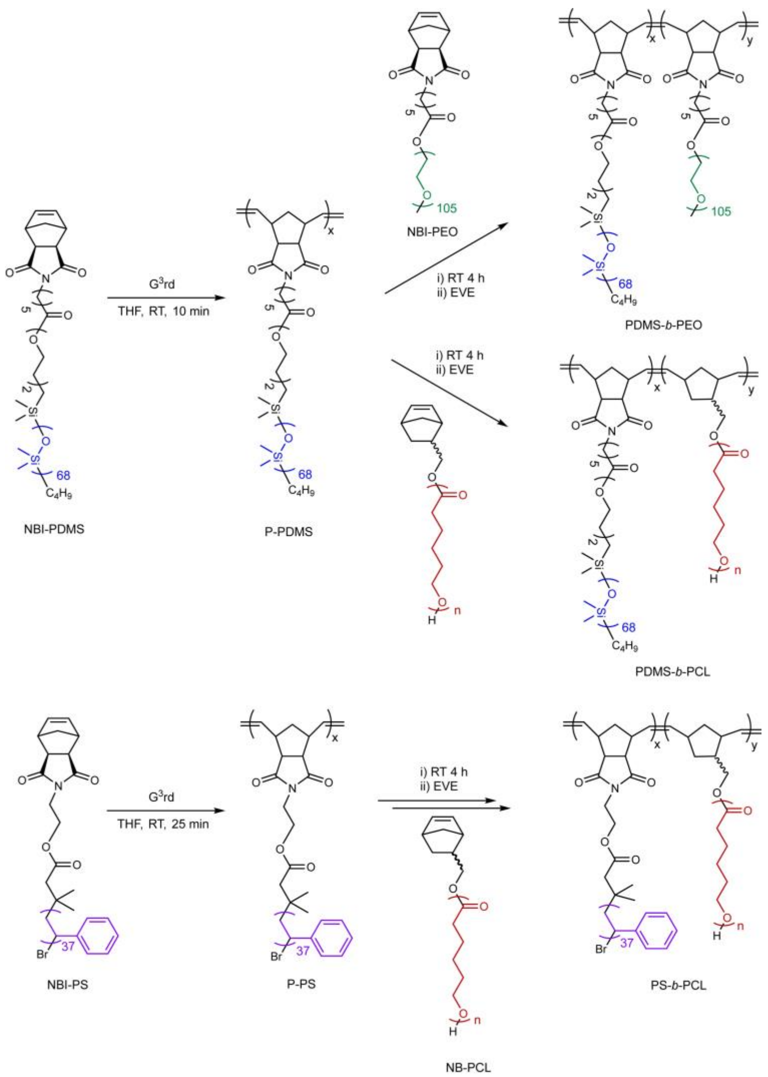

Supplementary Fig. 4. **Synthetic routes of BBCPs.** A schematic illustration showing the two-step grafting-through ROMP strategy for synthesizing BBCPs including PDMS-*b*-PEO, PDMS-*b*-PCL, and PS-*b*-PCL. The first macromonomer (NBI-PDMS or NBI-PS) was first polymerized via ROMP, followed by sequential addition of second macromonomer (NBI-PEO or NB-PCL).

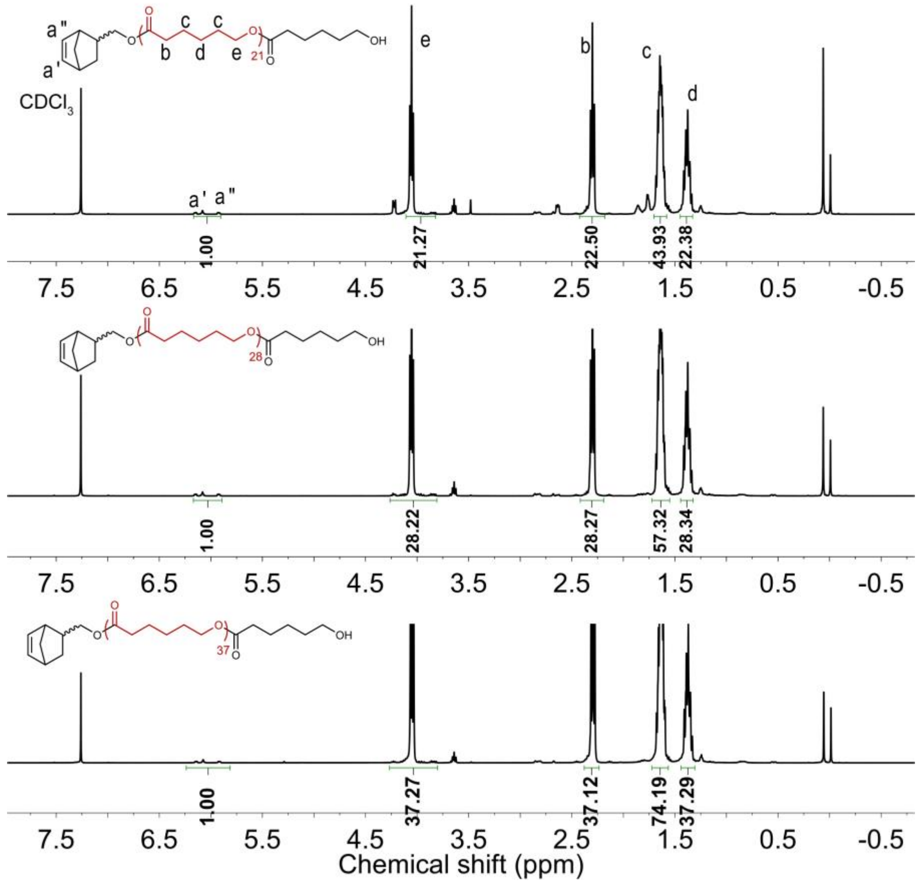

Supplementary Fig. 5. ^{1}H NMR spectra (400 MHz, $CDCl_3$) of NB-PCL with different molecular weights shows the presence of norbornene double bond (peaks a′, a″) and PCL repeating units (peaks b-e). The peak area ratios between of PCL repeating units and double bond are used to present the degree of polymerization of PCL.

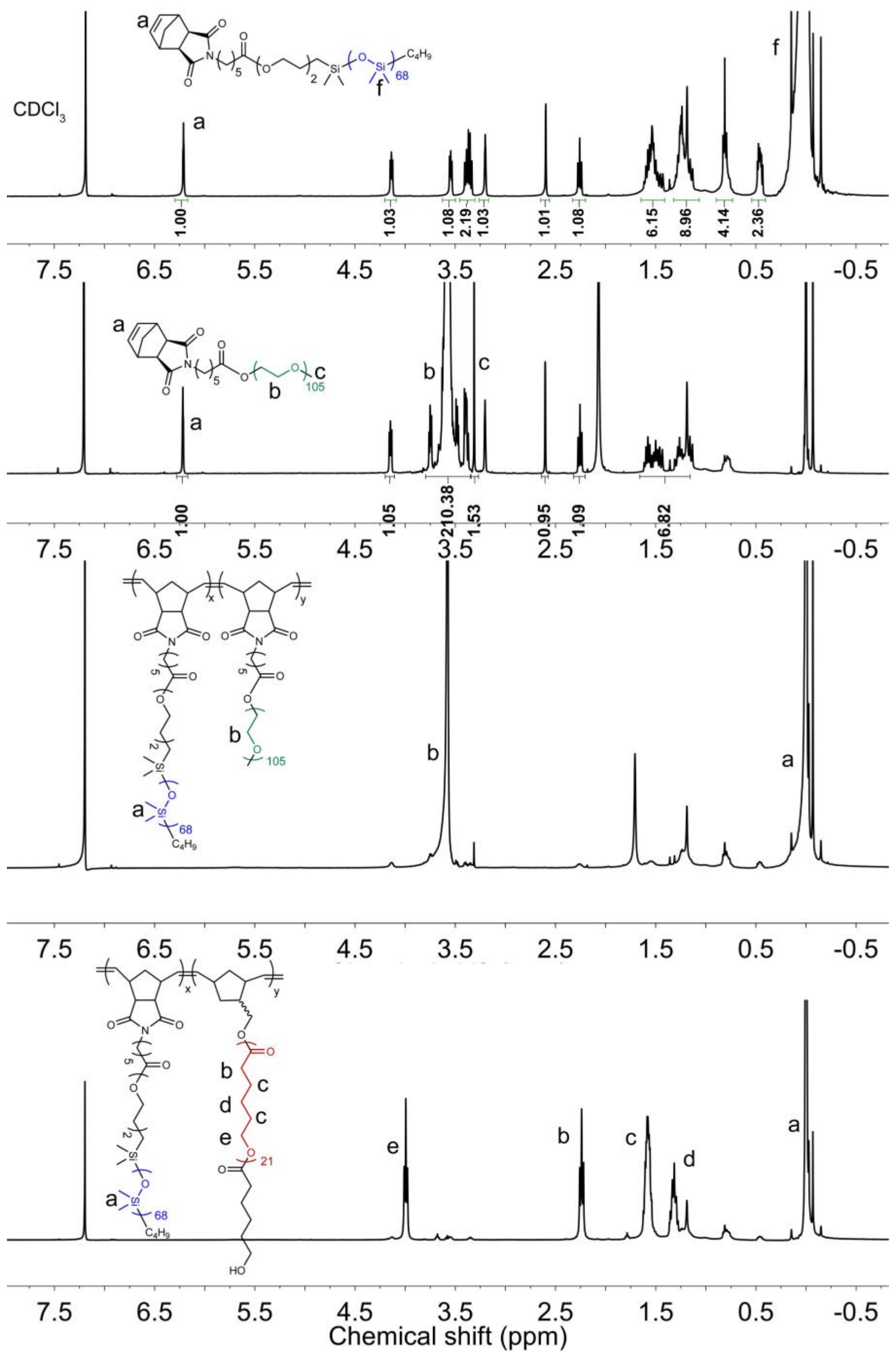

Supplementary Fig. 6. ^{1}H NMR spectra (400 MHz, $CDCl_3$) of NBI-PDMS$_{68}$, NBI-PEO$_{105}$, PDMS$_{68}^{150}$-46%PEO$_{105}^{132}$, and PDMS$_{68}^{110}$-42%PCL$_{22}^{182}$. Both PDMS and PEO macromonomers contain norbornene double bond (peak a) with PDMS repeating units (peak f) and PEO repeating units (peak b). PDMS-*b*-PEO contains both PDMS repeating units (peak a) and PEO repeating units (peak b). PDMS-*b*-PCL contains both PDMS (peak a) and PCL (peaks b-e) repeating units.

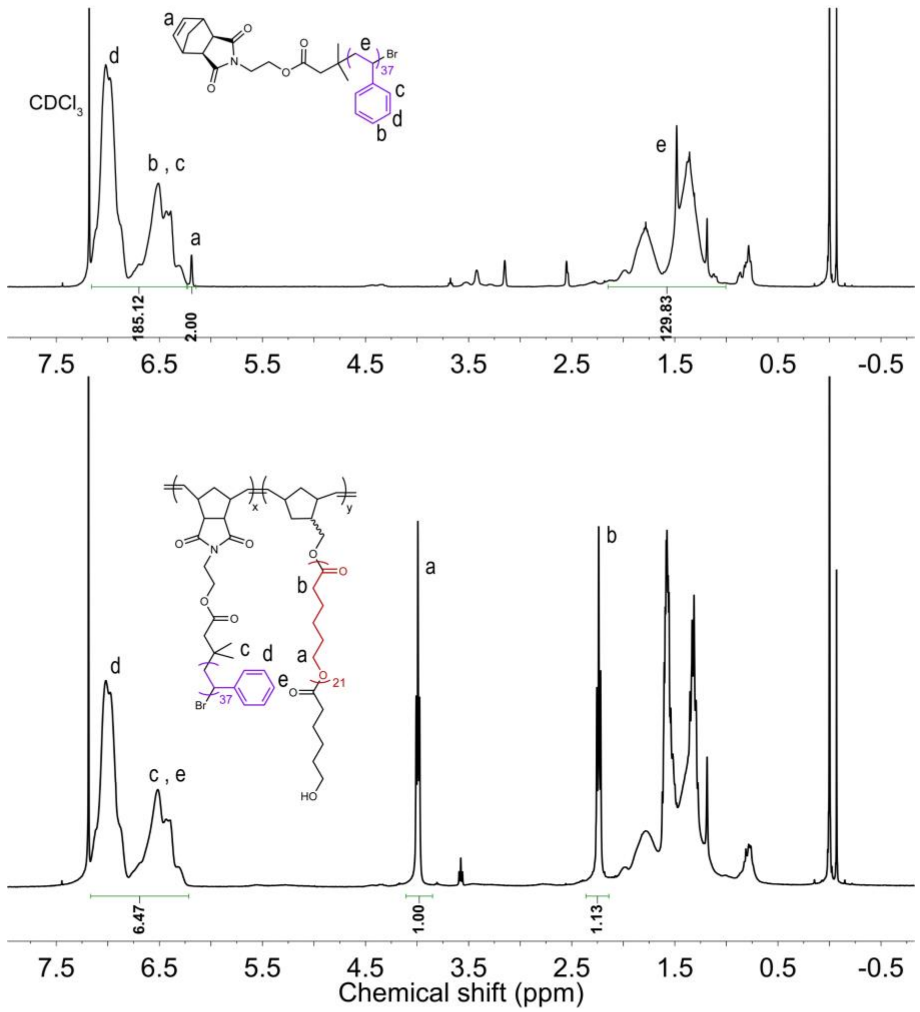

Supplementary Fig. 7. ^{1}H NMR spectra (400 MHz, $CDCl_3$) of NBI-PS_{37} and PS^{110}_{37}-40%PCL^{113}_{22}. NBI-PS_{37} contains aryl repeating units (peaks b, c, d, e). PS-*b*-PCL contains both PCL repeating units (peak a, b) and PS aryl repeating units (peak c, d, e, f).

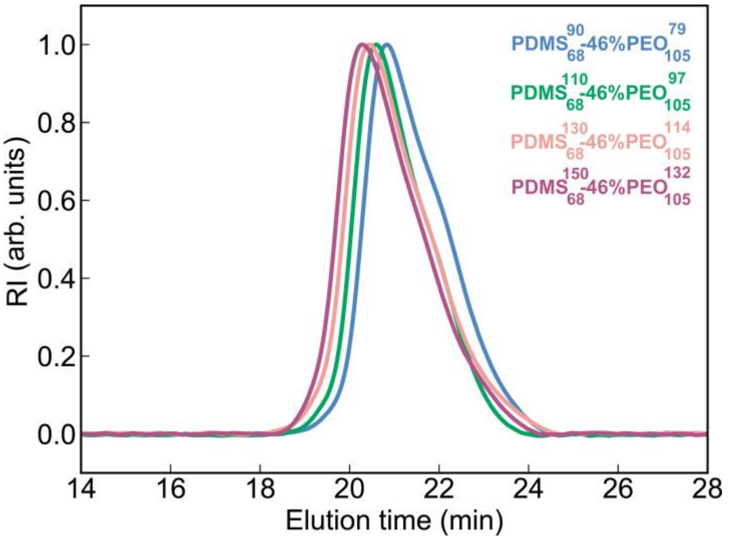

Supplementary Fig. 8. GPC curves of PDMS-*b*-PEO BBCPs with different molecular weights.

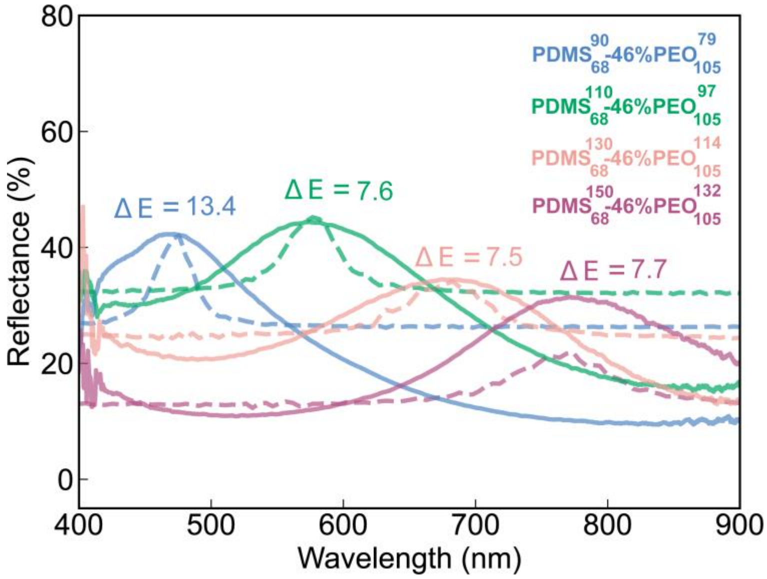

Supplementary Fig. 9. Simulated spectra based on the modified multilayer optical model (dotted line) match the experimental spectra taken by a microspectrometer (solid line) at the crystalline state. The color difference (ΔE) between simulated and experimental spectra are noted aside the spectra.

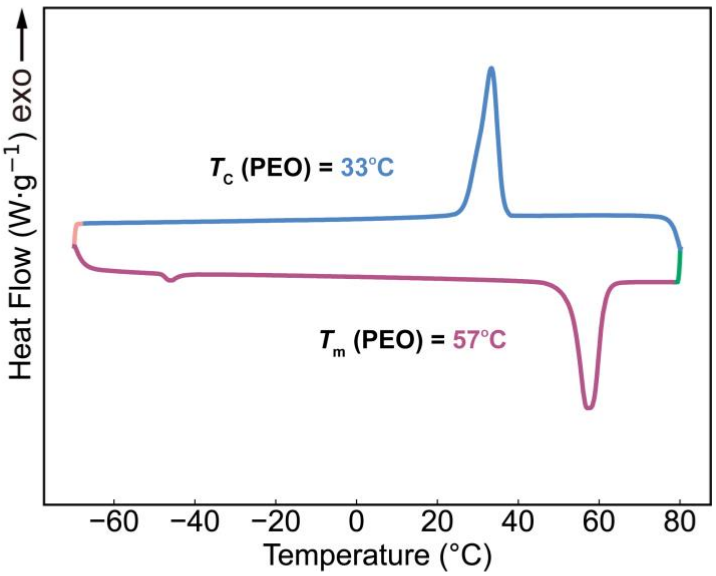

Supplementary Fig. 10. DSC curves of PDMS-*b*-PEO BBCPs with heating and cooling rates of 10°C min^{-1} after removing the thermal history.

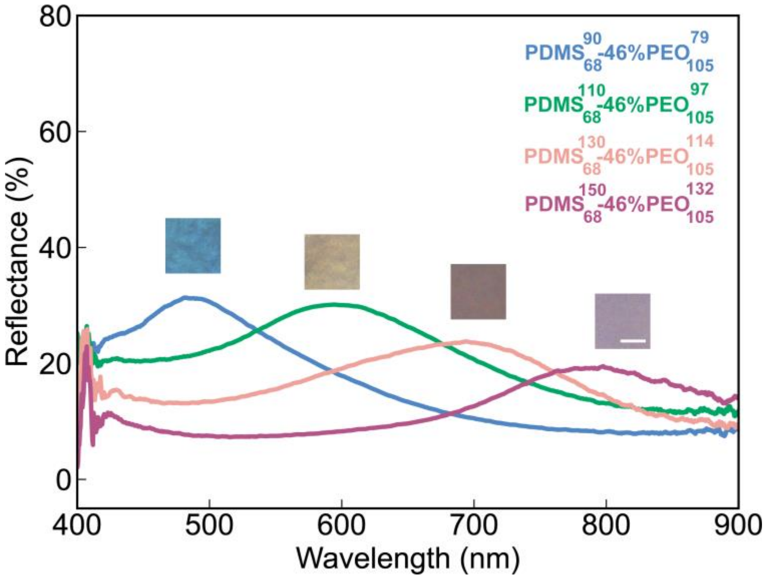

Supplementary Fig. 11. Reflectance spectra taken by a microspectrometer and bright field optical images of PDMS-*b*-PEO photonic films with different molecular weights at the melt state. Scale bars are 50 μm in optical images.

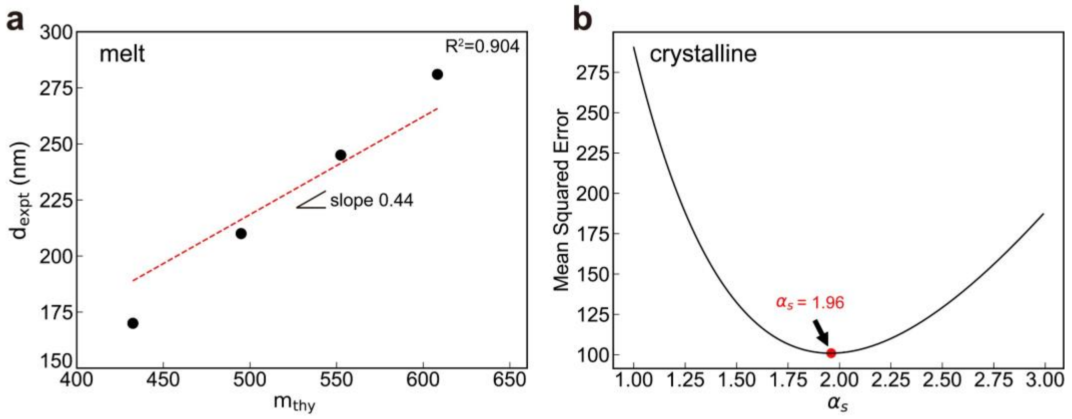

Supplementary Fig. 12. a, Linear fit between the experimentally measured spacing d_{expt} at the melt state and the theoretically calculated m_{thy} for four PDMS-*b*-PEO. The fit yields a slope of 0.44 with R^2=0.904. **b,** Minimization of the mean squared error between the predicted and measured domain spacing in the crystallized state determines the stiffening parameter of the side chain (α_s) for four PDMS-*b*-PEO. The measured domain spacing in the crystallized state is calculated from Supplementary Fig. 9.

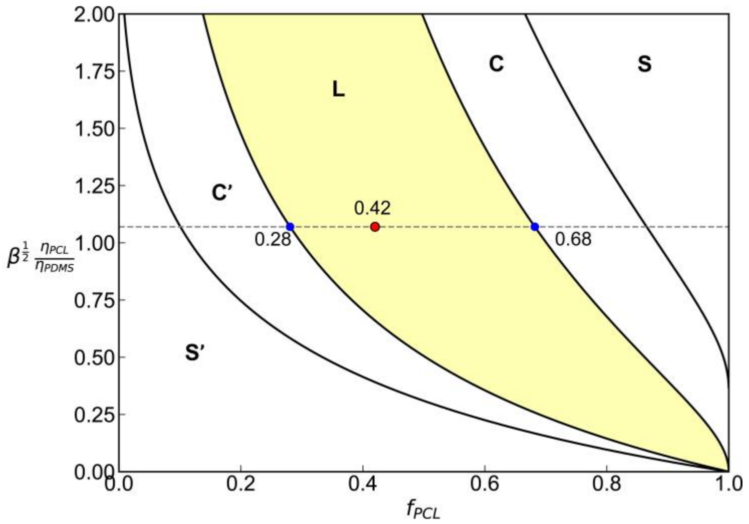

Supplementary Fig. 13. Theoretical phase diagram of PDMS-*b*-PCL with $n_{s,\mathrm{PDMS}} = 68$, $n_{s,\mathrm{PCL}} = 29$ at the crystallized state based on SS-SCF theory, including 1D lamellar (*L*), 2D cylindrical (*C, C'*), and 3D spherical phases (*S, S'*). The blue points mark the boundary volume fraction to form a lamellar phase, and the red point is the volume fraction we choose to make, which represent all BBCPs with various molecular weights at f_{PCL}=42%.

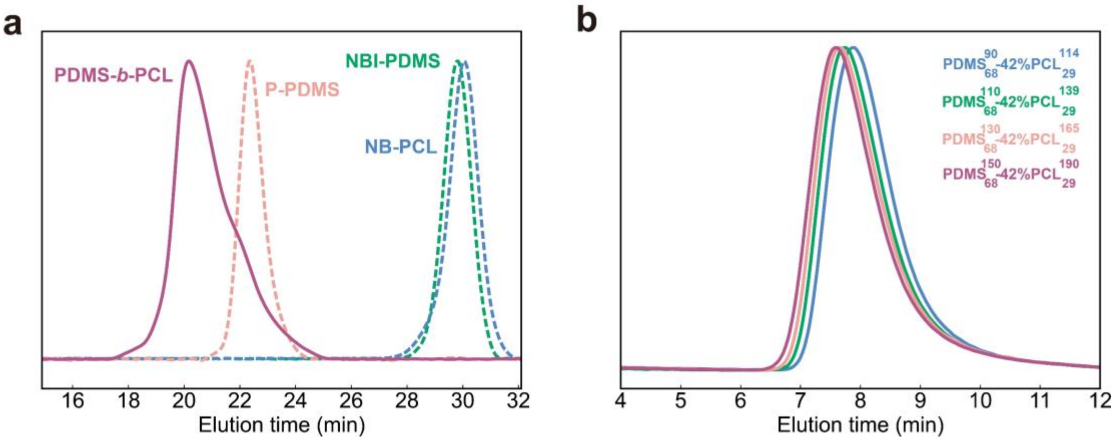

Supplementary Fig. 14. GPC curves of macromonomers and BBCPs. **a**, Comparisons of GPC spectra of PDMS-*b*-PCL, bottlebrush homopolymer (P-PDMS), PDMS macromonomer (NBI-$PDMS_{68}$), and PCL macromonomer (NB-PCL_{29}). **b**, PDMS-*b*-PCL BBCPs with different molecular weights and fixed PCL volume fraction (42%).

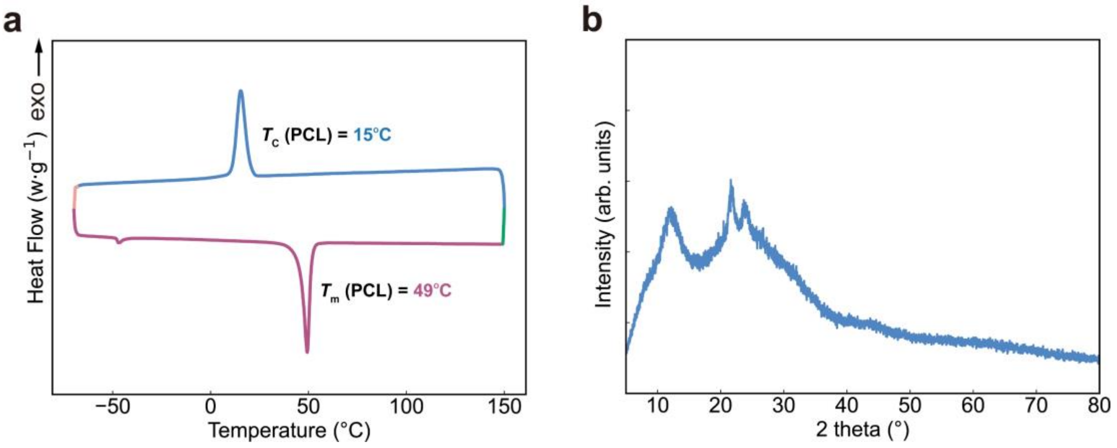

Supplementary Fig. 15. **a,** DSC curves of $PDMS_{68}^{110}$-42%PCL_{38}^{113} with heating and cooling rates of 10°C min^{-1} after removing thermal history. **b,** XRD curve of $PDMS_{68}^{110}$-42%PCL_{38}^{113} photonic film shows the presence of crystallization.

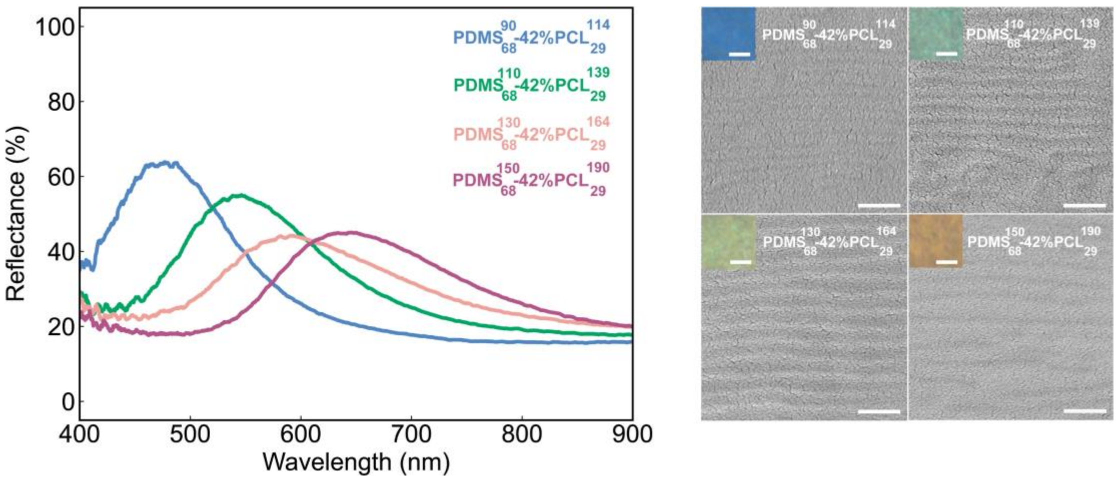

Supplementary Fig. 16. Reflectance spectra taken by an integrating sphere, bright field optical images, and cross-sectional SEM images of PDMS-*b*-PCL BBCPs photonic films with different molecular weights and fixed PCL volume fraction (42%). Scale bars are 500 nm in SEM images and 50 μm in optical images.

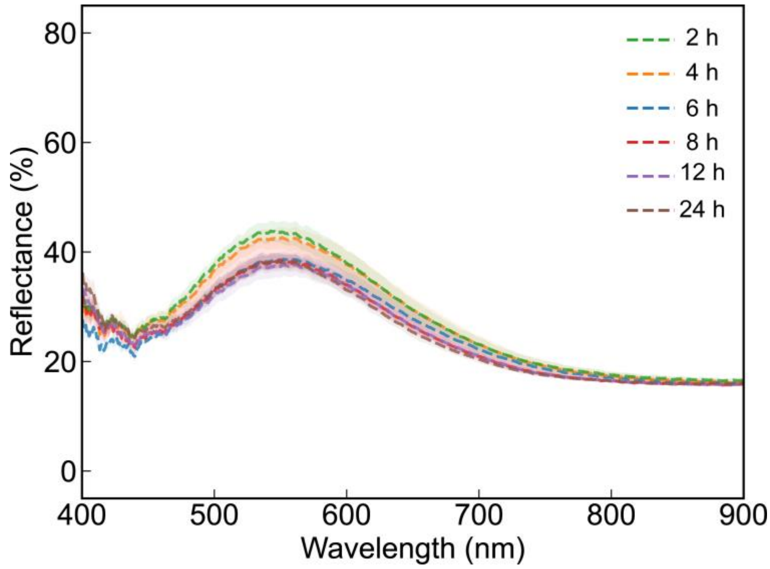

Supplementary Fig. 17. Reflectance spectra taken by an integrating sphere of $PDMS_{68}^{110}$-42%PCL_{29}^{139} films with different annealing time. The dashed line denotes the averaged reflectance spectrum of five measurements at different locations of one sample and the shaded area represents the standard deviation from five measurements.

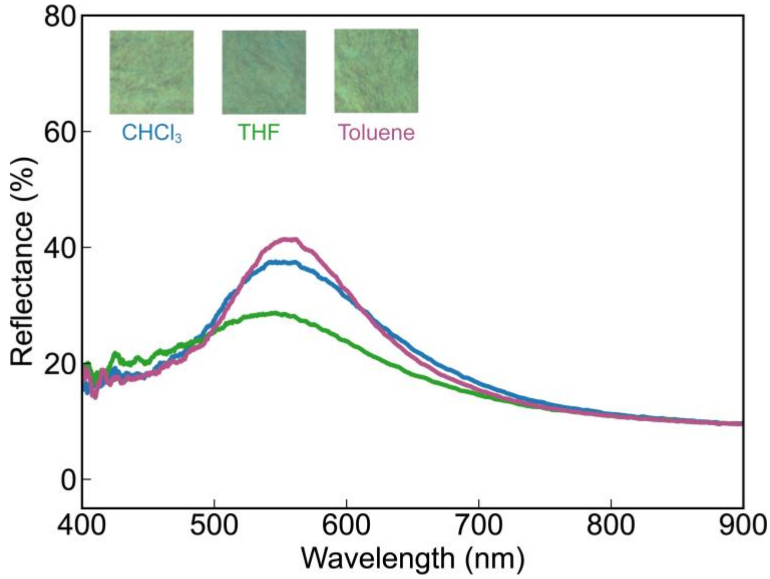

Supplementary Fig. 18. Reflectance spectra taken by an integrating sphere and bright field optical images of $PDMS_{68}^{110}$-42%PCL_{29}^{139} photonic films via different solvent vapor annealing. Insets are optical images with size of 0.25 cm × 0.25 cm.

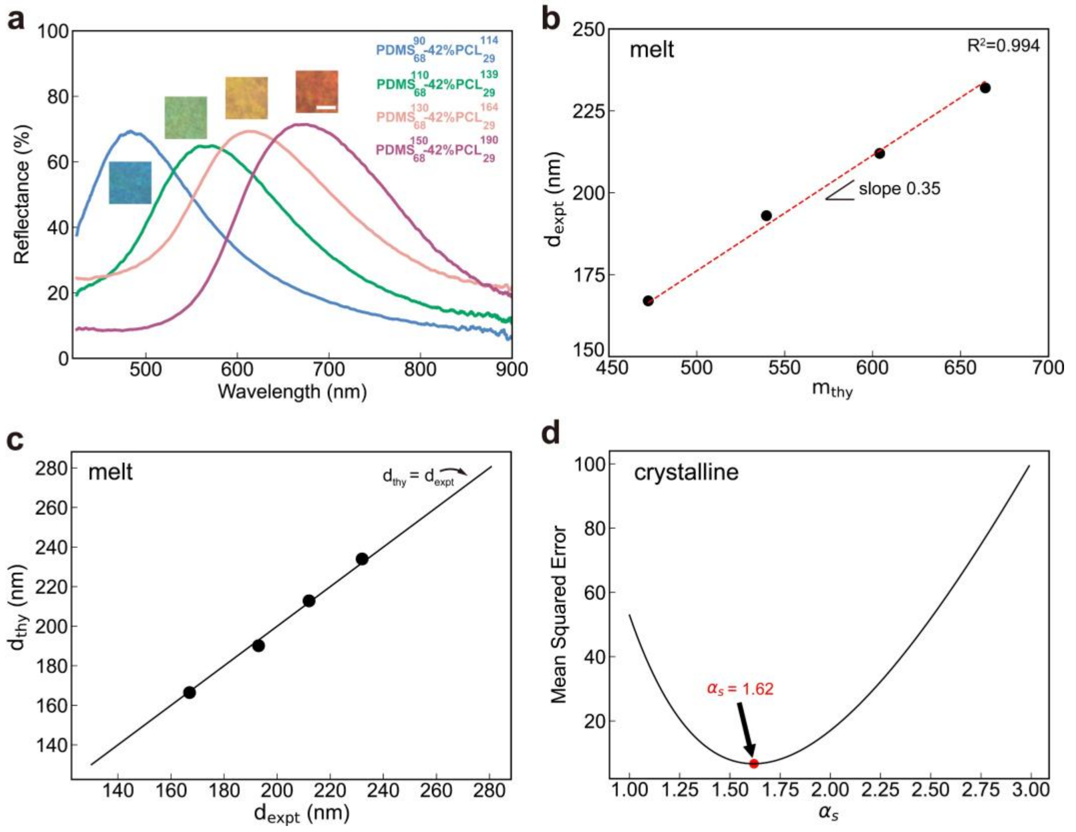

Supplementary Fig. 19. Optimization of $\tilde{L}_{\mathrm{A}}$ and α_{s} using four PDMS-*b*-PCL samples. **a,** Reflectance spectra taken by a microspectrometer and bright field optical images of PDMS-*b*-PCL photonic films with different molecular weights and fixed PCL volume fraction (42%) at the melt state. Scale bars are 50 μm in optical images. **b,** Linear fit between the measured spacing d_{expt} at the melt state and the calculated m_{thy} for four PDMS-*b*-PCL samples. The fit yields a slope of 0.35 with R^2=0.994. **c,** Theoretical and experimental domain spacings almost collapse on a master line for four PDMS-*b*-PCL BBCPs with different molecular weights at the melt state. **d,** Minimization of the mean squared error between the predicted and measured domain spacing in the crystallized state determines the stiffening parameter of the side chain (α_{s}) for four PDMS-*b*-PCL samples. The measured domain spacing in the crystallized state is calculated from Supplementary Fig. 16.

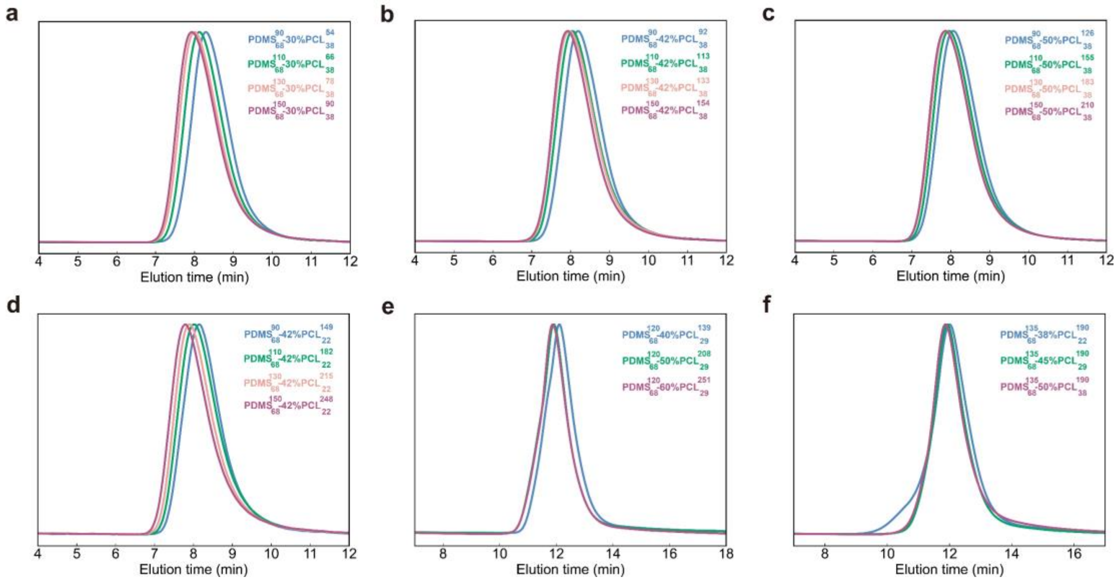

Supplementary Fig. 20. GPC curves of a series of PDMS-*b*-PCL BBCPs with various molecular structures: **a-c**, different PCL volume fractions (f%=30%, 42%, 50%) with $n_{s,\mathrm{PCL}}$= 38, **d,** different backbone lengths with f%=30% and $n_{s,\mathrm{PCL}}$= 22. **e,** different PCL volume fractions (f%= 40%, 50%, 60%) with $n_{s,\mathrm{PCL}}$= 29. **f,** different PCL side chain lengths ($n_{s,\mathrm{PCL}}$= 22, 29, 38) with the same polymerization degree of the backbone.

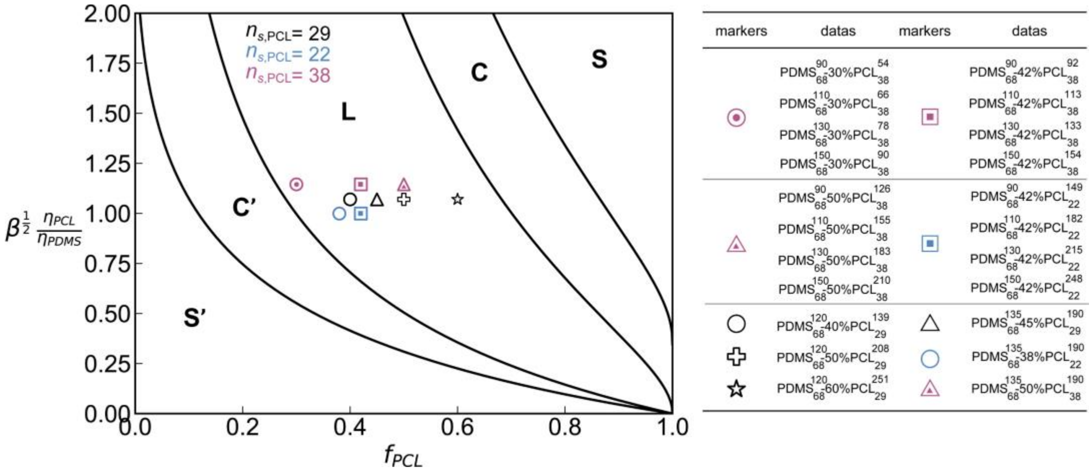

Supplementary Fig. 21. Theoretical phase diagram of PDMS-*b*-PCL BBCPs at the crystallized state based on our modified SS-SCF theory. The data points consist of 22 PDMS-*b*-PCL samples with different chain architectures and they all fall within the lamellar phase region, matching experimental observations. Some samples with same volume fraction and side chain length occupy the same position in the phase diagram.

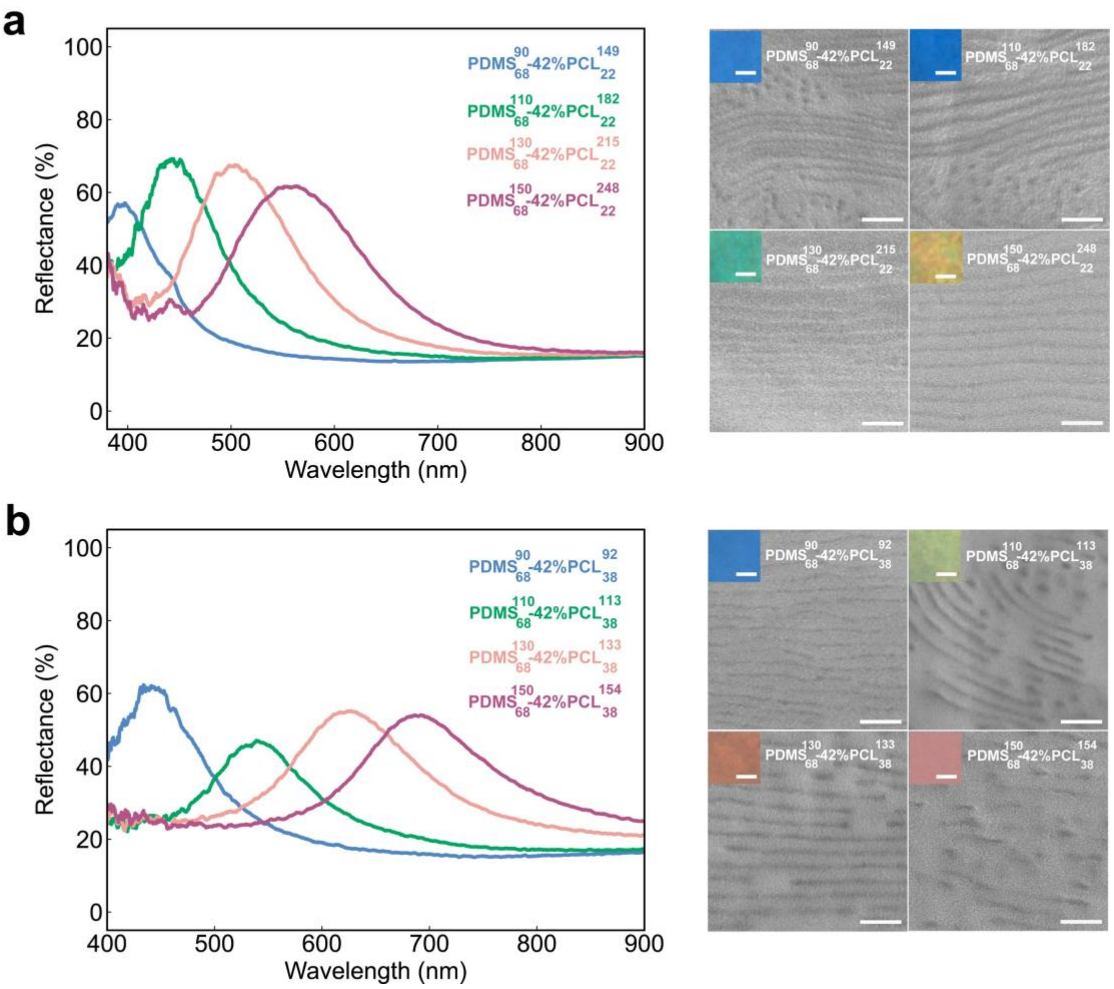

Supplementary Fig. 22. Reflectance spectra taken by an integrating sphere, bright field optical images, and cross-sectional SEM images of PDMS-*b*-PCL BBCPs photonic films with different molecular weights, PCL side chain lengths and fixed PCL volume fraction (f%= 42%). **a,** Samples with $n_{s,\mathrm{PCL}}$= 22. **b,** Samples with $n_{s,\mathrm{PCL}}$= 29. Scale bars are 500 nm in SEM images and 50 μm in optical images.

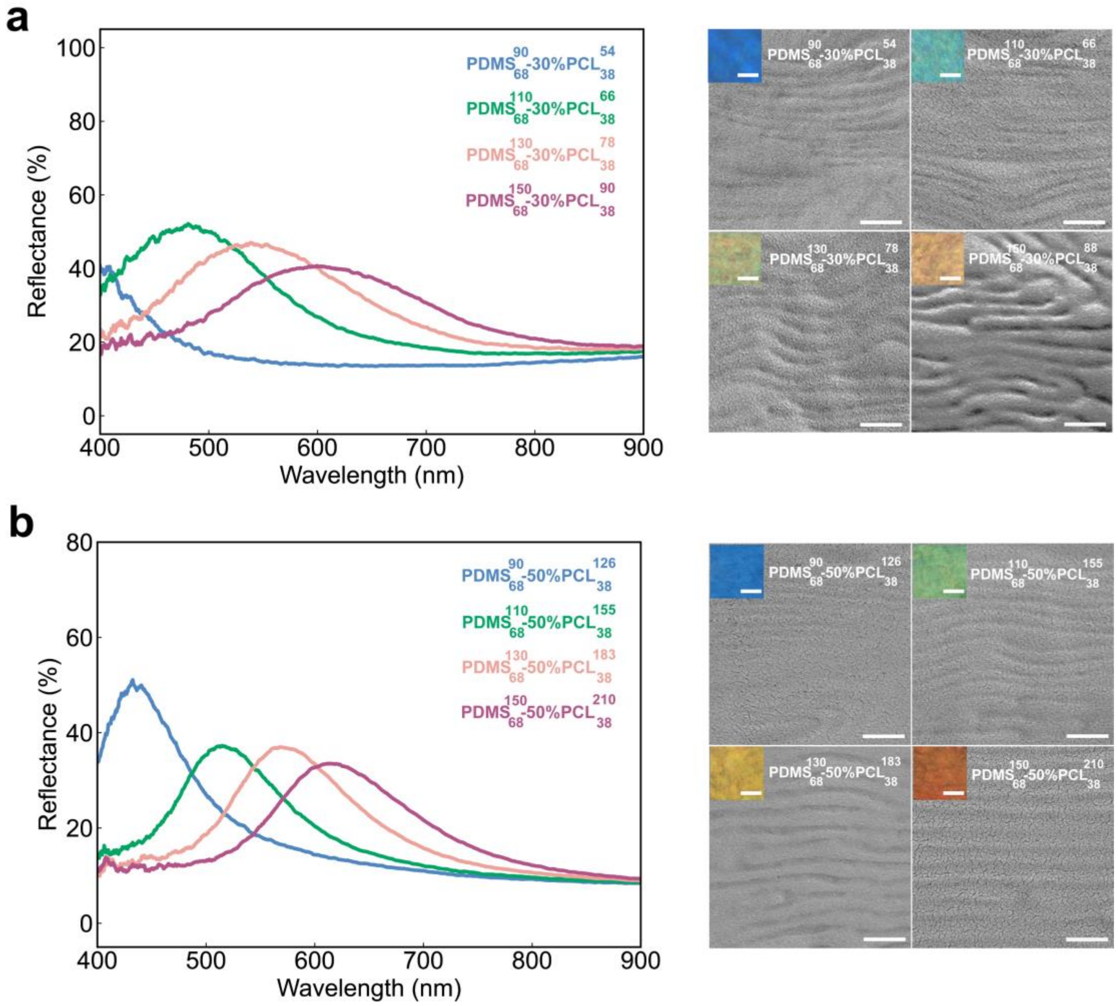

Supplementary Fig. 23. Reflectance spectra taken by an integrating sphere, bright field optical images, and cross-sectional SEM images of PDMS-*b*-PCL BBCPs photonic films with different PCL volume fraction and fixed PCL side chain length ($n_{s,\mathrm{PCL}}$= 38). **a,** Samples with $f\% = 30\%$. **b,** Samples with $f\% = 50\%$. Scale bars are 500 nm in SEM images and 50 μm in optical images.

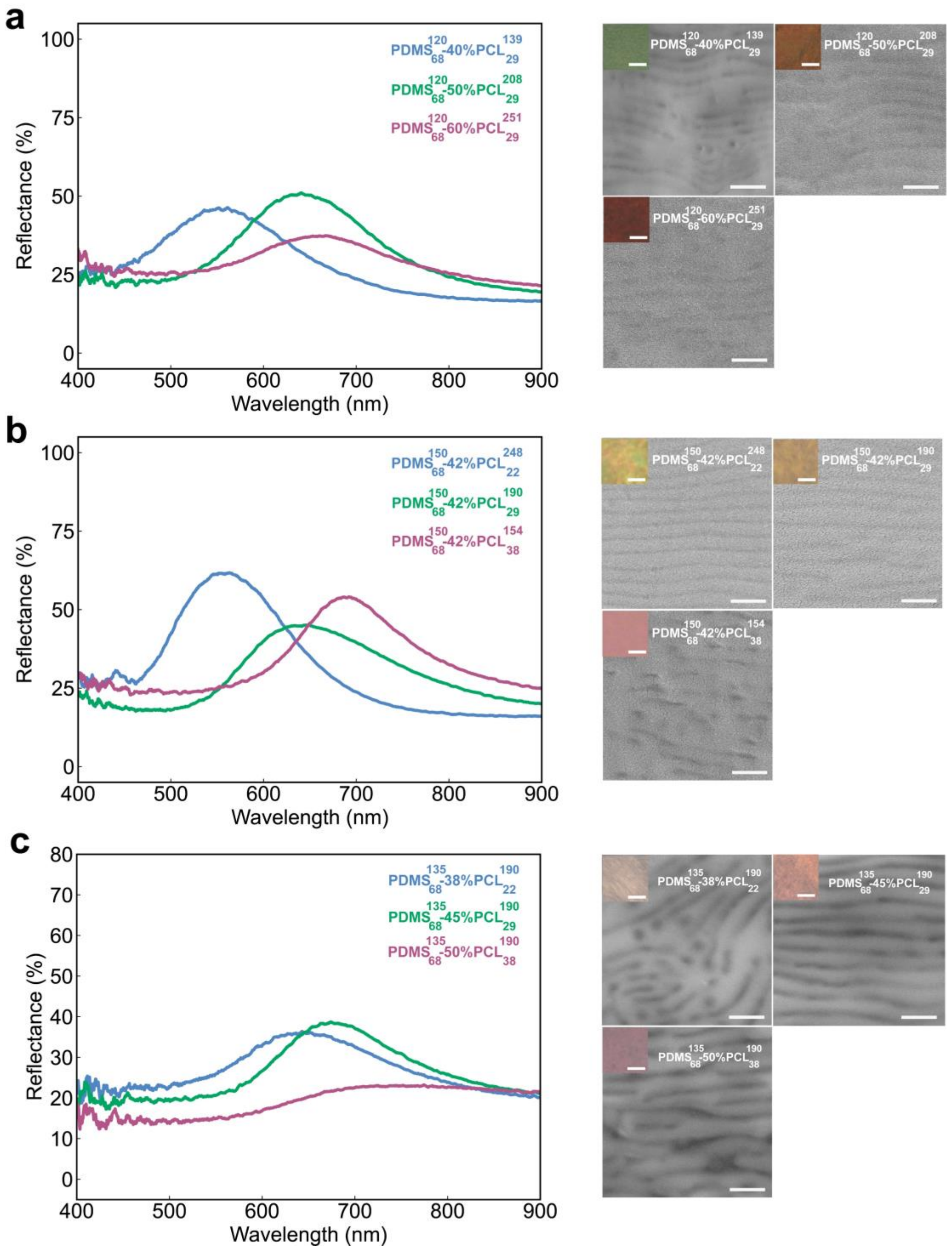

Supplementary Fig. 24. Reflectance spectra taken by an integrating sphere, bright field optical images, and cross-sectional SEM images of PDMS-*b*-PCL BBCPs photonic films with different molecular parameters. **a,** Samples with different PCL volume fractions ($f\% = 40\%$, 50%, 60%) and fixed $n_{s,\text{PCL}}$= 29. **b,** Samples with different PCL side chain lengths ($n_{s,\text{PCL}}$= 22, 29, 38) and fixed $f\% = 42\%$. **c,** Samples with different side chain lengths ($n_{s,\text{PCL}}$= 22, 29, 38) and fixed backbone degree of polymerization (N= 325). Scale bars are 500 nm in SEM images and 50 μm in optical images.

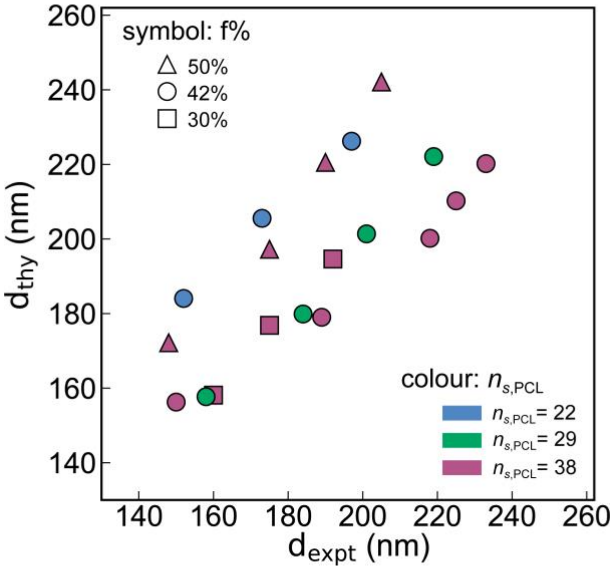

Supplementary Fig. 25. Theoretical and experimental domain spacings almost collapse on a master line for 19 PDMS-*b*-PCL BBCPs (including 15 data points and four reference data points) with varying backbone lengths ($n_{b,\mathrm{PDMS}}$, $n_{b,\mathrm{PCL}}$) and PCL side-chain length ($n_{s,\mathrm{PCL}}$) and PCL volume fractions (f%) at the melt state.

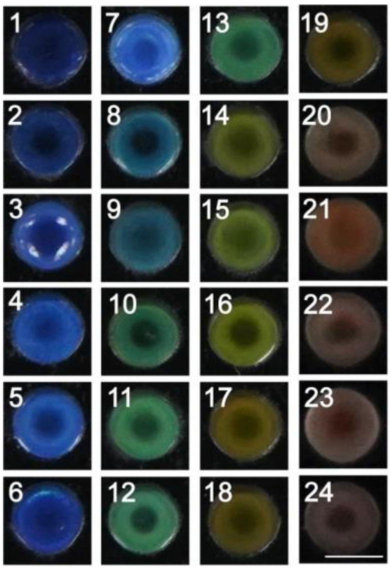

1	$\mathrm{PDMS}_{68}^{90}-30\%\mathrm{PCL}_{38}^{54}$	13	$\mathrm{PDMS}_{68}^{130}-30\%\mathrm{PCL}_{38}^{78}$
2	$\mathrm{PDMS}_{68}^{90}-42\%\mathrm{PCL}_{22}^{149}$	14	$\mathrm{PDMS}_{68}^{110}-42\%\mathrm{PCL}_{38}^{113}$
3	$\mathrm{PDMS}_{68}^{90}-42\%\mathrm{PCL}_{38}^{92}$	15	$\mathrm{PDMS}_{68}^{150}-42\%\mathrm{PCL}_{22}^{248}$
4	$\mathrm{PDMS}_{68}^{90}-50\%\mathrm{PCL}_{38}^{126}$	16	$\mathrm{PDMS}_{68}^{138}-38\%\mathrm{PCL}_{22}^{190}$
5	$\mathrm{PDMS}_{68}^{110}-42\%\mathrm{PCL}_{22}^{182}$	17	$\mathrm{PDMS}_{68}^{150}-42\%\mathrm{PCL}_{29}^{190}$
6	$\mathrm{PDMS}_{68}^{90}-42\%\mathrm{PCL}_{29}^{142}$	18	$\mathrm{PDMS}_{68}^{130}-42\%\mathrm{PCL}_{38}^{133}$
7	$\mathrm{PDMS}_{68}^{110}-30\%\mathrm{PCL}_{38}^{66}$	19	$\mathrm{PDMS}_{68}^{120}-60\%\mathrm{PCL}_{29}^{251}$
8	$\mathrm{PDMS}_{68}^{110}-42\%\mathrm{PCL}_{29}^{139}$	20	$\mathrm{PDMS}_{68}^{130}-50\%\mathrm{PCL}_{38}^{183}$
9	$\mathrm{PDMS}_{68}^{130}-42\%\mathrm{PCL}_{22}^{215}$	21	$\mathrm{PDMS}_{68}^{150}-42\%\mathrm{PCL}_{38}^{154}$
10	$\mathrm{PDMS}_{68}^{110}-50\%\mathrm{PCL}_{38}^{155}$	22	$\mathrm{PDMS}_{68}^{135}-45\%\mathrm{PCL}_{29}^{190}$
11	$\mathrm{PDMS}_{68}^{130}-42\%\mathrm{PCL}_{29}^{164}$	23	$\mathrm{PDMS}_{68}^{135}-50\%\mathrm{PCL}_{38}^{190}$
12	$\mathrm{PDMS}_{68}^{120}-40\%\mathrm{PCL}_{29}^{139}$	24	$\mathrm{PDMS}_{68}^{150}-50\%\mathrm{PCL}_{38}^{210}$

Supplementary Fig. 26. Colour swatches produced by assembling BBCPs with various chain architectures. Scale bar, 1.5 mm.

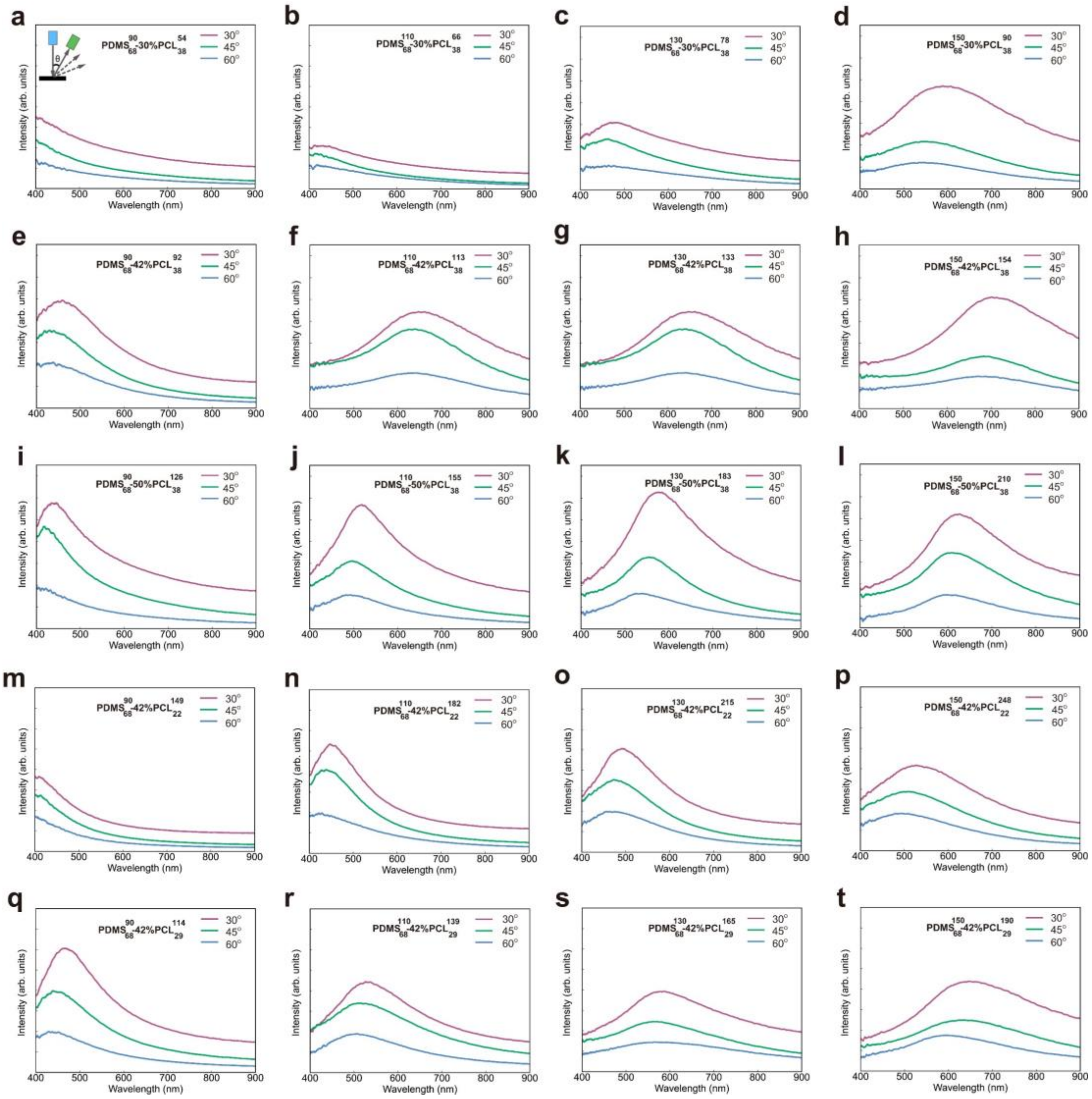

Supplementary Fig. 27. Angle-resolved scattering spectra of PDSM-*b*-PCL photonic films with different molecular structures. The incident light is normal to the sample surface and the detection angle varies from 30° to 60°.

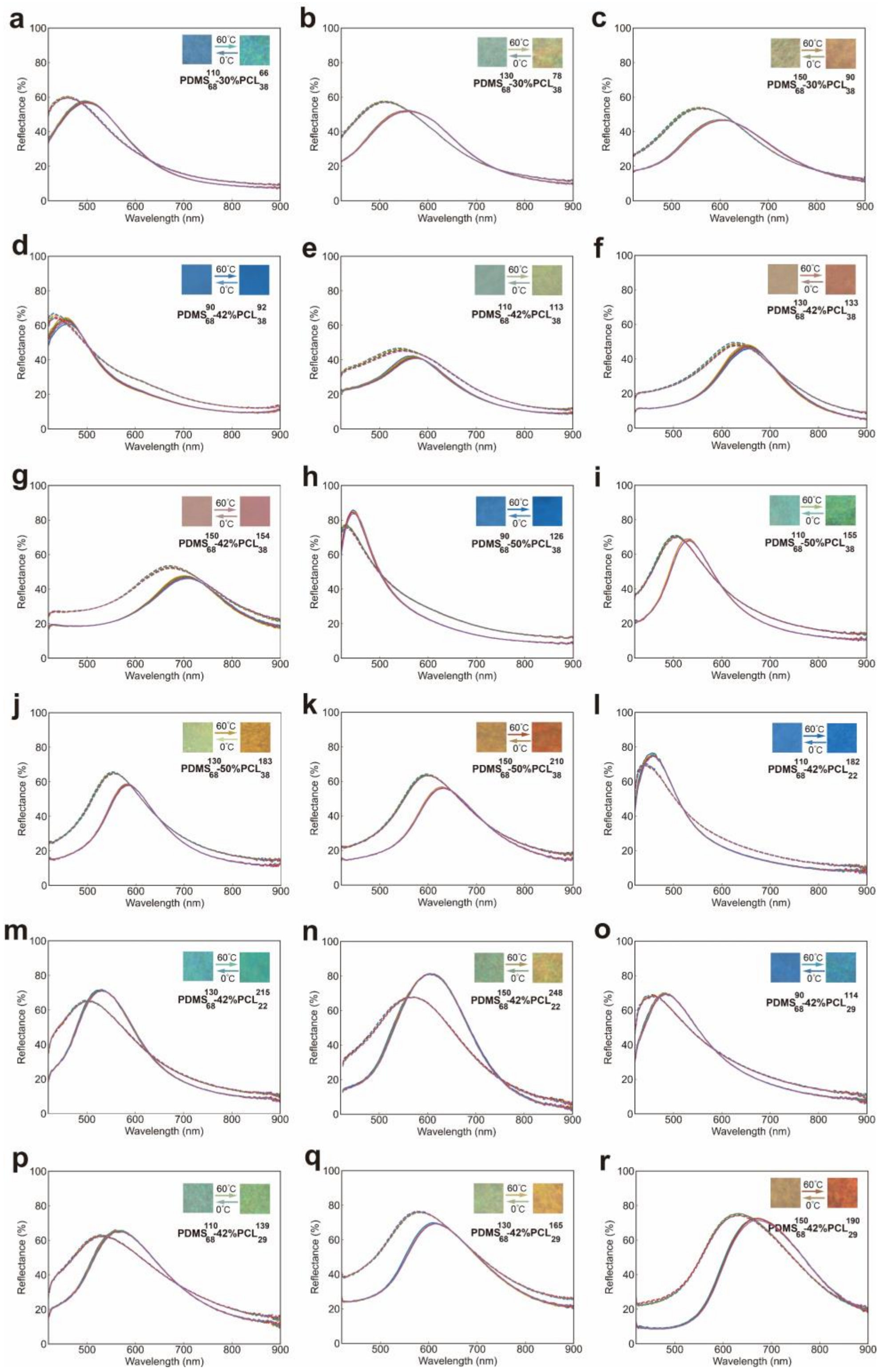

Supplementary Fig. 28. Reflectance spectra taken by a microspectrometer of PDMS-*b*-PCL photonic films with different molecular structures during five heating-cooling cycles (optical images size: 0.25 cm × 0.25 cm). Solid and dash lines represent equilibrated spectra at 60°C and 0°C. The five colours (blue, orange, green, red, purple) correspond to five sequential cycles (cycle 1 to cycle 5).

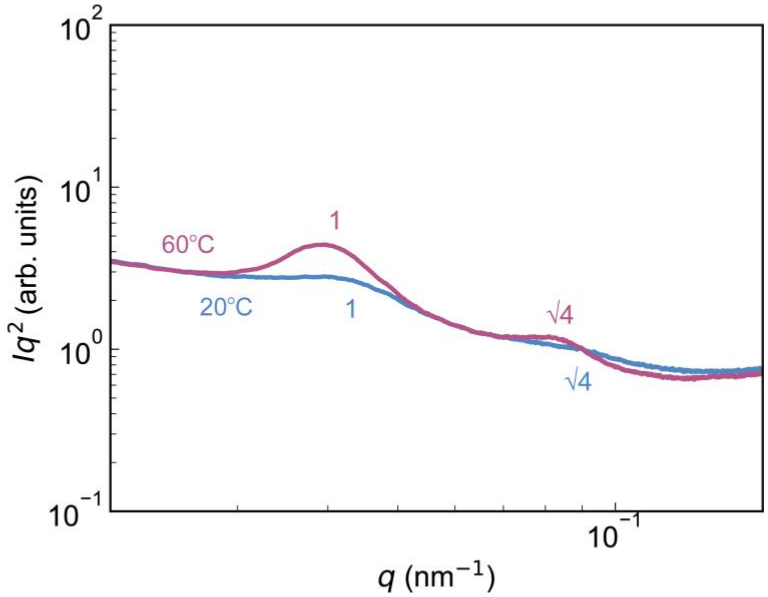

Supplementary Fig. 29. USAXS profiles of representative $PDMS_{68}^{90}$-50%PCL_{38}^{126} film equilibrated at 20°C and 60°C.

Detailed analysis of d-spacing is provided below.

d-spacing (nm)	20°C	60°C
USAXS	145.5[a]	156.3[a]
λ_{peak}	148.1[b]	153.2[b]

[a] The d-spacing is calculated by equation $2\pi/q$.[b] The d-spacing is calculated by Bragg's law from the reflectance peak position.

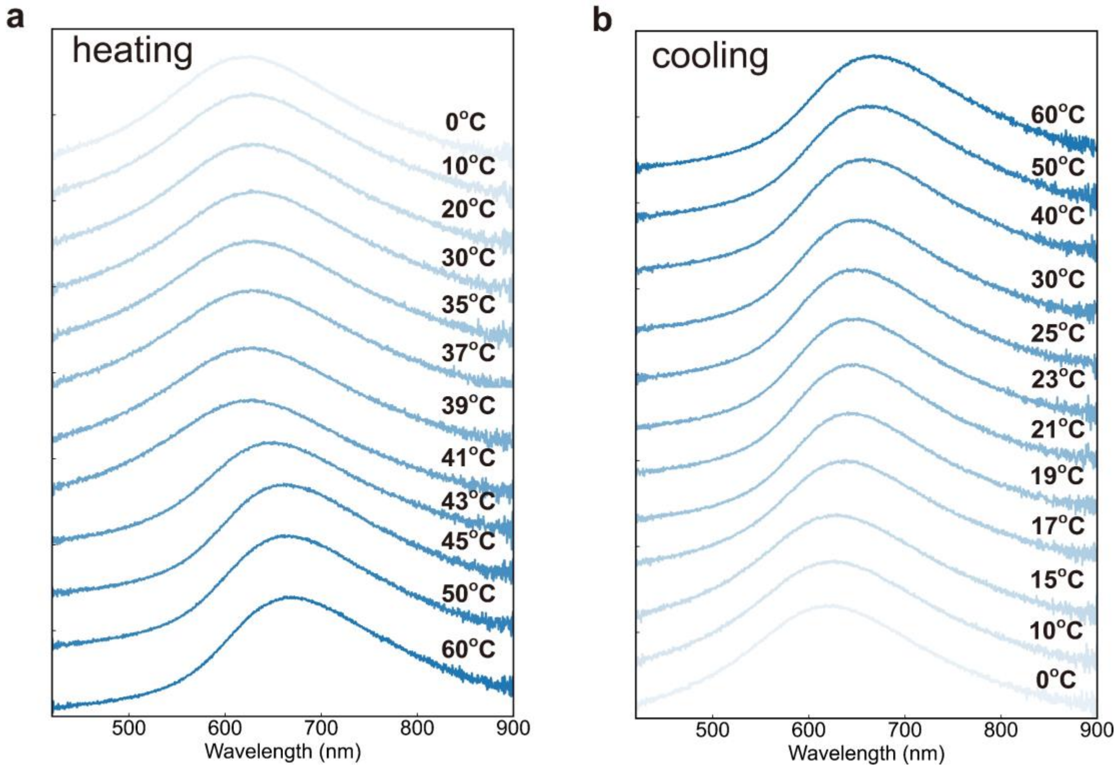

Supplementary Fig. 30. *In-situ* reflectance spectra taken by a microspectrometer with temperature for $PDMS_{68}^{150}$-42%PCL_{29}^{190} at a heating/cooling rate of 10°C min^{-1}: **a**, heating process; **b**, cooling process.

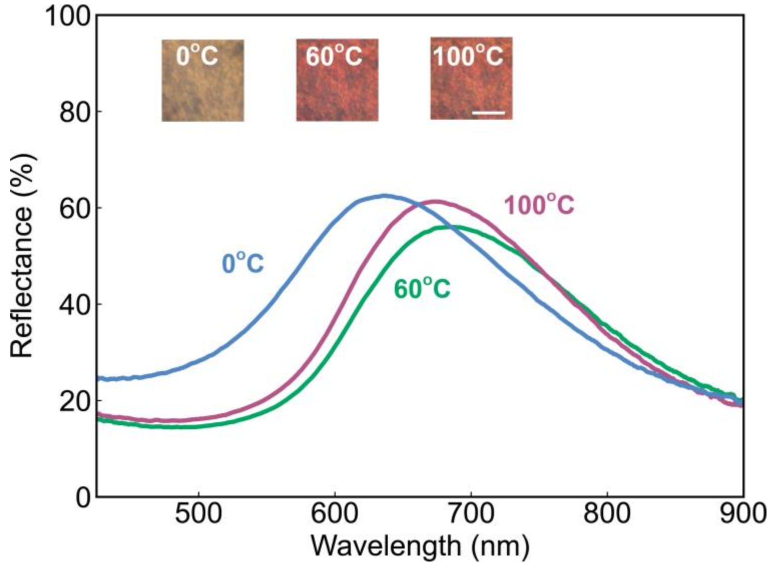

Supplementary Fig. 31. Reflectance spectra taken by a microspectrometer of $PDMS_{68}^{150}$-42%PCL_{29}^{190} film equilibrated at 0°C, 60°C, and 100°C. Insets are optical images with scale bars of 100 μm.

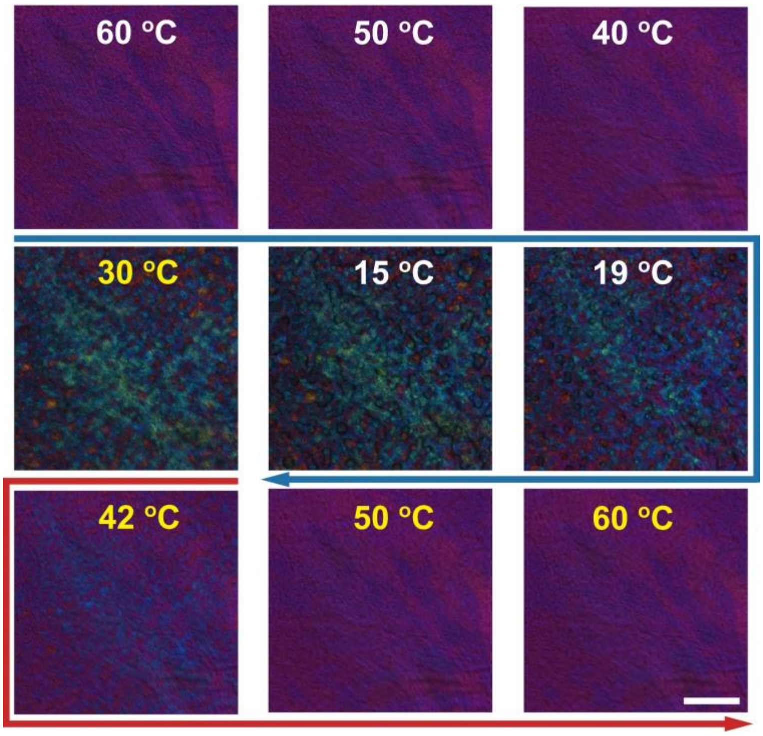

Supplementary Fig. 32. POM images of $PDMS_{68}^{150}$-42%PCL_{29}^{190} photonic film with temperature at a cooling (blue line) and heating (red line) rate of 10°C min^{-1}. Insets are optical images with scale bars of 50 μm.

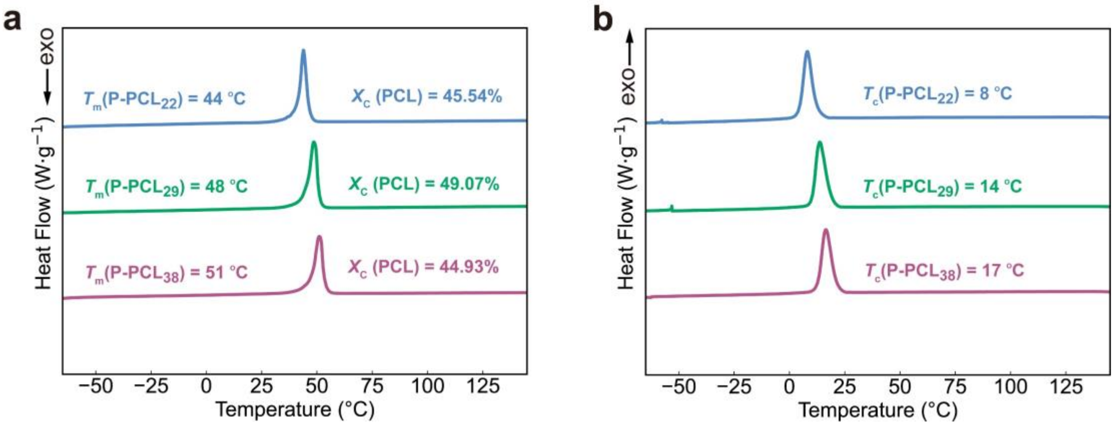

Supplementary Fig. 33. DSC curves of homo bottlebrush (P-PCL) with the heating/cooling rate of 10°C min^{-1}: **a**, the heating process after removing thermal history at 150°C; and **b**, the cooling process.

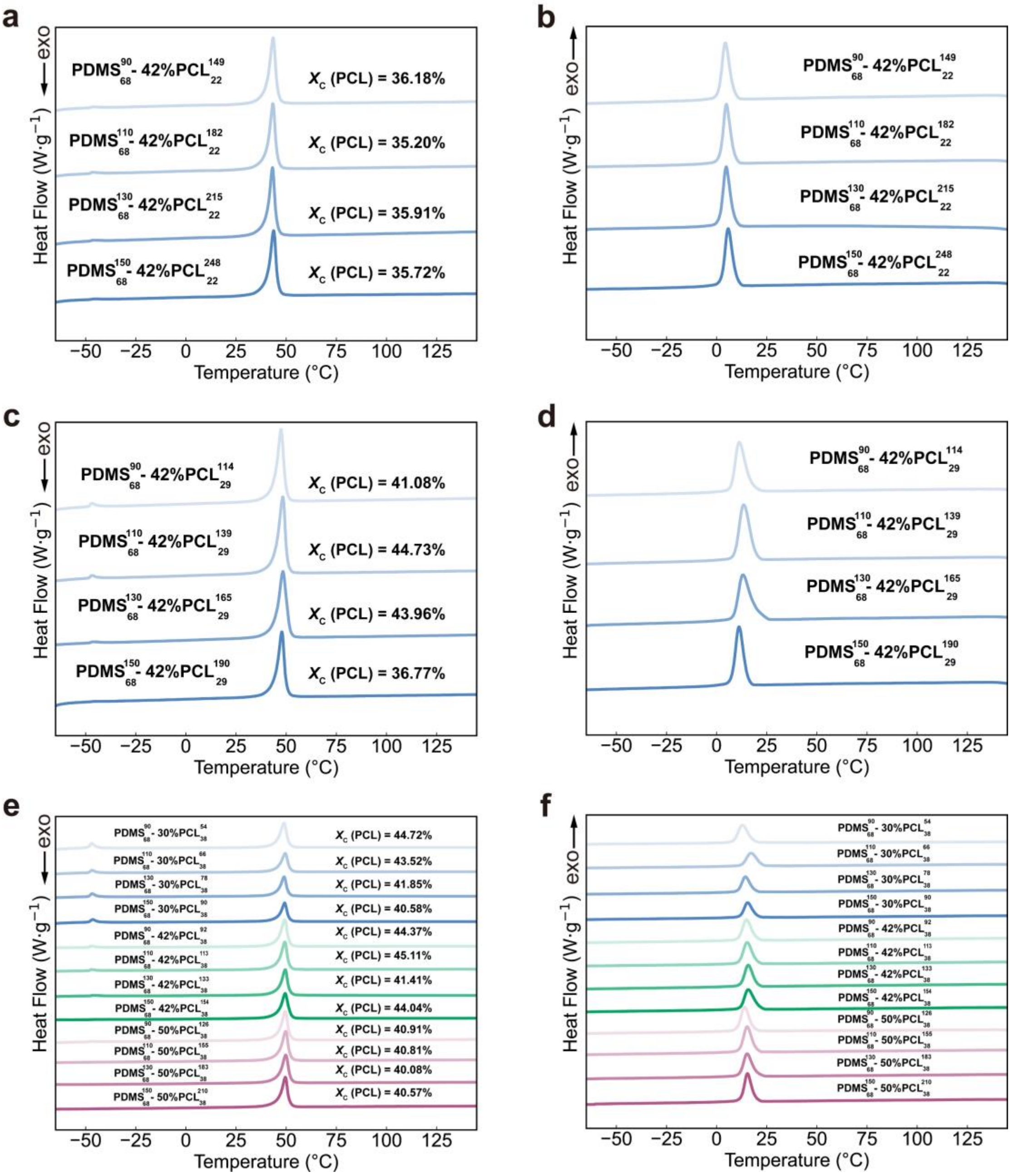

Supplementary Fig. 34. DSC curves of PDMS-*b*-PCL BBCPs with different molecular structures with the heating/cooling rate of 10℃ min^{-1}. **a, c, e**, The heating process after removing thermal history at 150℃. **b, d, f**, The cooling process.

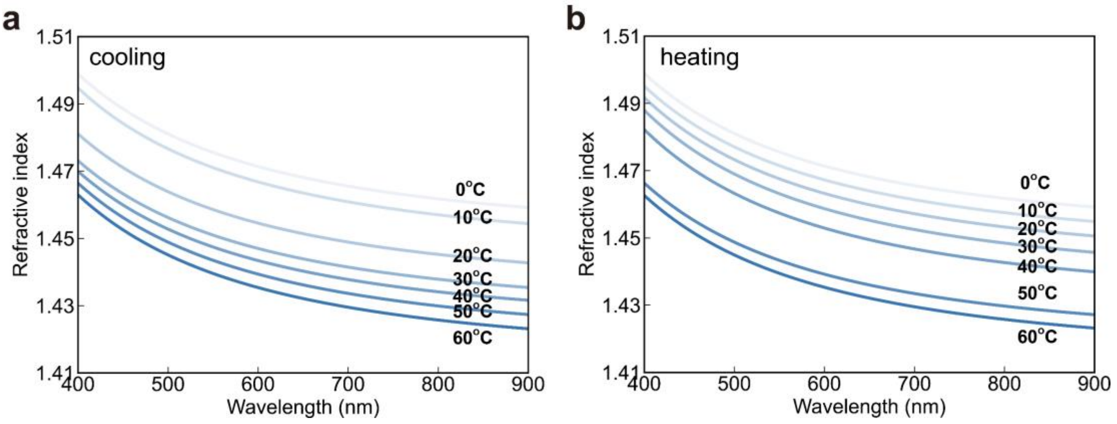

Supplementary Fig. 35. Wavelength-dependent refractive index of spin-cast $PDMS_{68}^{150}$-42%PCL_{29}^{190} film with the heating/cooling rate of 10°C min^{-1}. Each data point is obtained under testing conditions within 20 seconds at the corresponding temperature.

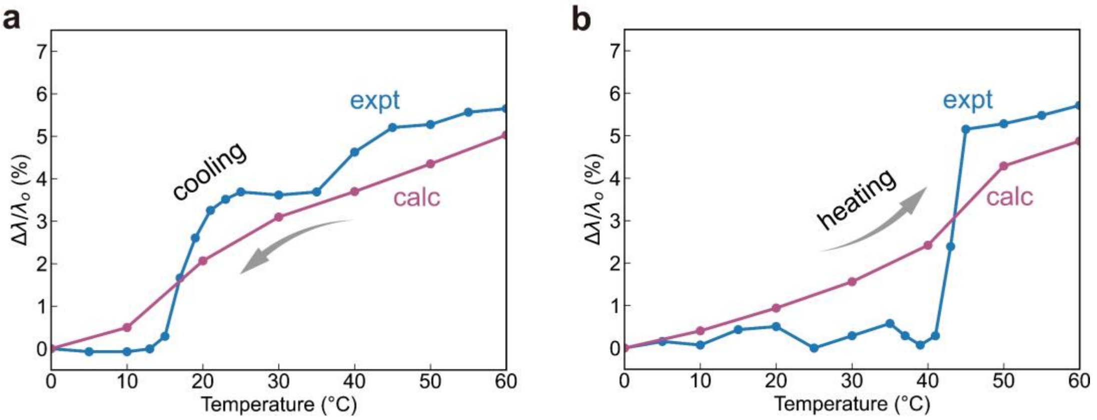

Supplementary Fig. 36. Reflectance peak shifts with temperature from experimental measurements and calculations based on ellipsometry results during heating and cooling. $\Delta\lambda = \lambda_{\text{heat}} - \lambda_{\text{cool}}$ represents the peak shift upon thermal cycling. The sample is produced by assembling $PDMS_{68}^{150}$-42%PCL_{29}^{190}.

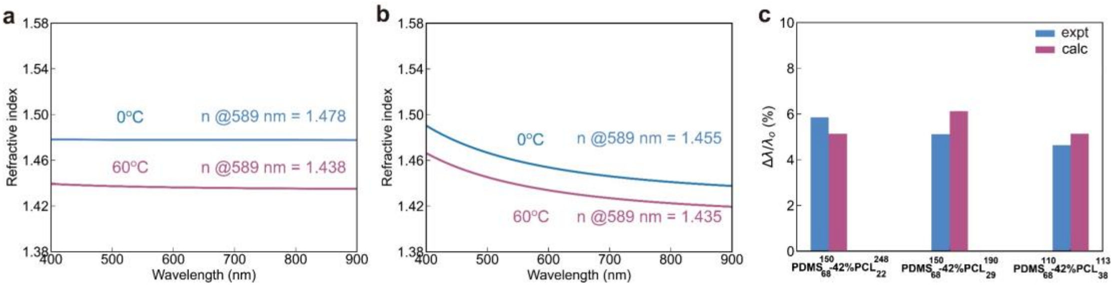

Supplementary Fig. 37. The refractive index changes and the comparison of scattering peak shift ratios ($\Delta\lambda/\lambda_o$) between experimental results with ellipsometer results of photonic film with different chain architectures at 0°C and 60°C. **a-b,** The wavelength-dependent refractive index at 0°C and 60°C for (**a**) $PDMS_{68}^{150}$-42%PCL_{22}^{248} and (**b**) $PDMS_{68}^{110}$-42%PCL_{38}^{113}. **c,** The comparison of scattering peak shift ratios ($\Delta\lambda/\lambda_o$) between measured spectra and calculated spectra based on ellipsometer results at 0°C and 60°C.

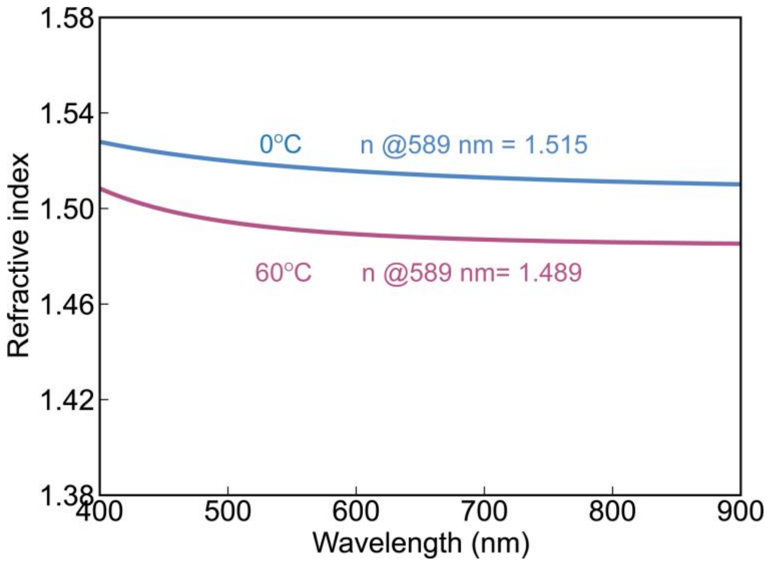

Supplementary Fig. 38. The refractive index changes of P-PCL film at 0°C and 60°C.

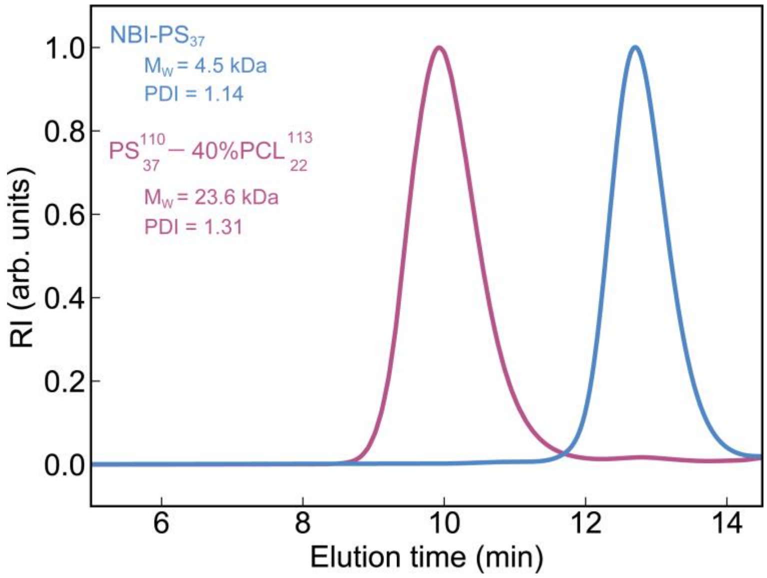

Supplementary Fig. 39. GPC curves of NBI-PS_{37} and PS_{37}^{110}-40%PCL_{22}^{113}

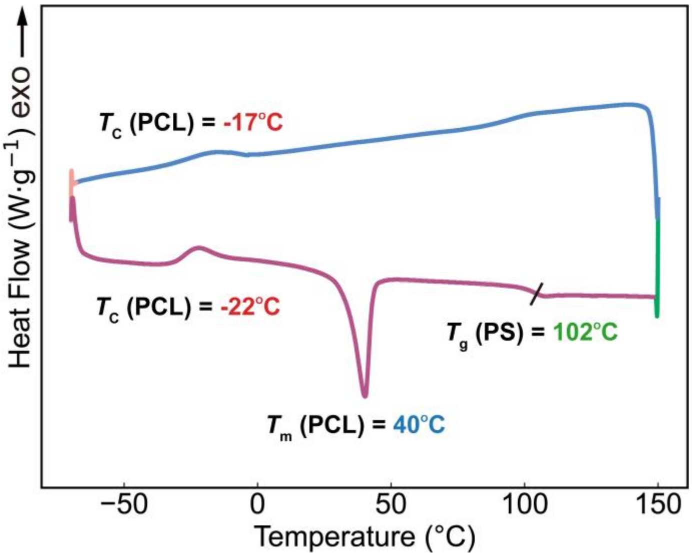

Supplementary Fig. 40. DSC curves of PS_{37}^{110}-40%PCL_{22}^{113} after removing thermal history with heating and cooling rates of 10°C min^{-1}.

References

1. Zhulina, E. B., Sheiko, S. S., Dobrynin, A. V. & Borisov, O. V. Microphase segregation in the melts of bottlebrush block copolymers. *Macromolecules* **53**, 2582–2593 (2020).
2. Liang, H., Wang, Z., Sheiko, S. S. & Dobrynin, A. V. Comb and bottlebrush graft copolymers in a melt. *Macromolecules* **52**, 3942–3950 (2019).
3. Liang, H. *et al.* Universality of the entanglement plateau modulus of comb and bottlebrush polymer melts. *Macromolecules* **51**, 10028–10039 (2018).
4. Wagner, M. & Wolf, B. A. Effect of block copolymers on the interfacial tension between two 'immiscible' homopolymers. *Polymer* **34**, 1460–1464 (1993).
5. Bersenev, E. A., Nikitina, E. A., Dashtimoghadam, E., Sheiko, S. S. & Ivanov, D. A. Bottlebrush elastomers with crystallizable side chains: monitoring configuration of polymer backbones in the amorphous regions during crystallization. *ACS Macro Lett.* **11**, 1085–1090 (2022).
6. Bruggeman, D. A. G. Berechnung verschiedener physikalischer konstanten von heterogenen substanzen. I. dielektrizitätskonstanten und leitfähigkeiten der mischkörper aus isotropen substanzen. *Annalen der Physik* **416**, 636–664 (1935).
7. Miao, W. *et al.* An orthogonal dynamic covalent polymer network with distinctive topology transformations for shape- and molecular architecture reconfiguration. *Angew Chem Int Ed* **61**, e202109941 (2022).
8. Seong, H., Chen, Z., Emrick, T. & Russell, T. P. Reconfiguration and reorganization of bottlebrush polymer surfactants. *Angew Chem Int Ed* **61**, e202200530 (2022).
9. Reynolds, V. G. *et al.* Super-soft solvent-free bottlebrush elastomers for touch sensing. *Mater. Horiz.* **7**, 181–187 (2020).
10. Sveinbjörnsson, B. R. *et al.* Rapid self-assembly of brush block copolymers to photonic crystals. *Proc. Natl. Acad. Sci. U.S.A.* **109**, 14332–14336 (2012).
11. Gross, R. A. & Kalra, B. Biodegradable polymers for the environment. *Science* **297**, 803–807 (2002).
12. Cheng, G. *et al.* Conformation of oligo(ethylene glycol) grafted poly(norbornene) in solutions: a small angle neutron scattering study. *Eur. Polym. J.* **44**, 2859–2864 (2008).
13. *Hiemenz, P. C. & Lodge, T. P. Polymer Chemistry, 2nd Edn (Taylor & Francis, 2007).* (Taylor & Francis Group, 2007).
14. Kawai, A., Hamamoto, N. & Sasanuma, Y. Conformational characteristics and conformation-dependent properties of poly(ε-caprolactone). *Phys. Chem. Chem. Phys.* **24**, 11382–11394 (2022).
15. Jenkins, M. J. & Harrison, K. L. The effect of molecular weight on the crystallization kinetics of polycaprolactone. *Polymers for Advanced Techs* **17**, 474–478 (2006).
